# Computational Study of the Addition of Methanethiol to 40+ Michael Acceptors as a Model for the Bioconjugation of Cysteines

**DOI:** 10.1021/acs.joc.1c00349

**Published:** 2021-04-29

**Authors:** Anna M. Costa, Lluís Bosch, Elena Petit, Jaume Vilarrasa

**Affiliations:** Organic Chemistry Section, Facultat de Química, Universitat de Barcelona, Diagonal 645, Barcelona 08028, Catalonia, Spain

## Abstract

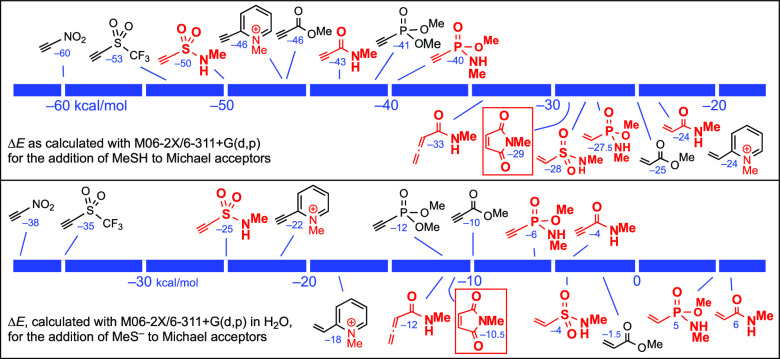

A long series of Michael acceptors are studied computationally as potential alternatives to the maleimides that are used in most antibody–drug conjugates to link Cys of mAbs with cytotoxic drugs. The products of the reaction of methanethiol (CH_3_SH/MeSH, as a simple model of Cys) with N-methylated ethynesulfonamide, 2-ethynylpyridinium ion, propynamide, and methyl ethynephosphonamidate (that is, with HC≡C–EWG) are predicted by the M06-2X/6-311+G(d,p) method to be thermodynamically more stable, in relation to their precursors, than that of MeSH with *N*-methylmaleimide and, in general, with H_2_C=CH–EWG; calculations with AcCysOMe and *^t^*BuSH are also included. However, for the addition of the anion (MeS^–^), which is the reactive species, the order changes and N-methylated 2-vinylpyridinium ion, 2,3-butadienamide, and maleimide may give more easily the anionic adducts than several activated triple bonds; moreover, the calculated Δ*G*^⧧^ values increase following the order HC≡C–SO_2_NHMe, *N*-methylmaleimide, HC≡C–PO(OMe)NHMe, and HC≡C–CONHMe. In other words, MeS^–^ is predicted to react more rapidly with maleimides than with ethynephosphonamidates and with propynamides, in agreement with the experimental results. New mechanistic details are disclosed regarding the advantageous use of some amides, especially of ethynesulfonamides, which, however, are more prone to double additions and exchange reactions.

## Introduction

Most antibody–drug conjugates (ADCs) on the market or in clinical development are prepared by the addition reaction of the sulfanyl or sulfhydryl groups of antibody cysteine units/residues (Cys) to a maleimide ring bound through a spacer to the cytotoxic drug, usually an antimitotic agent.^1^ The drawback with this process is that the addition products to maleimides (the adducts, which are succinimides) undergo quick, premature thiol exchanges in vivo, with glutathione and/or blood proteins, and are easily hydrolyzed.^2,3^ This instability of the thiol–maleimide bioconjugates is an important issue, which has been addressed in different ways, by the modification of the maleimide structure, either before the conjugation reaction or afterward,^2^ and by the search for alternative electron-withdrawing groups (EWGs).^4^ Double and triple bonds linked to strong EWGs (that is, good Michael acceptors) are in principle required, as relatively rapid additions are essential, bearing in mind that the couplings are usually carried out between biomolecules of high MW under very dilute physiological conditions. This work is mainly focused on the addition of thiols to double and triple bonds activated by one EWG, as compared to that to the double bond of maleimides. When the triple bond has two EWGs or when the double bond is substituted by two or more EWGs, the reactivity of the Michael acceptor may increase, but we will not deal systematically with these cases: apart from maleimides, maleic anhydride, and analogs, only a few examples are included at the beginning of this study for comparison. The important radical-initiated thiol–yne or thiol–ene reactions and the alkylation of thiolates with alkyl, allyl, or benzyl-like halides are not considered here either.

Six very recent reports are highly important with regard to the addition of Cys to (or conjugation with) Michael acceptors of the H_2_C=CH–EWG and HC≡C–EWG types.^5^ Chen et al.^5a^ used ethenesulfonamides, including *N*-phenyl derivatives functionalized at the *para* position; the reduced form of trastuzumab was attached to the reagent by means of a conjugate addition to the double bond. Our group^5b^ investigated the addition of Cys, glutathione, and reduced oxytocin to propynamides (HC≡C–CONHR) at 37 °C and pH 7.4, which exclusively yielded the resistant-to-exchange *Z* adducts (*trans* additions of RS^–^ and H^+^); we also compared the reaction rates of propynamides, propynoates, and maleimides. Hackenberger et al.^5c^ reported that phosphonamidates HC≡C–PO(OR)NHAr, where the aryl group was functionalized at the *para* position for linkage to biotin and fluorophores, gave rise to Cys-selective adducts, from reduced trastuzumab, also showing excellent stability to thiol exchange. Bernardes and coworkers^5d^ used quaternized 2-vinylpyridines and 2-ethynylpyridines, which exhibit a reactivity comparable to that of *N*-alkylmaleimides and much higher than that of 2-vinylpyridine or 2-ethynylpyridine, with the Cys residues of five different protein scaffolds (including Thiomab).^5d^ Winne et al.^5e^ published work on reversible dynamic exchanges of thioacetal linkages, that is, the double addition of thiols to several ynones, one propynamide, one ethynesulfonamide, and tosylacetylene (ethynyl 4-methylphenyl sulfone)^6^ in connection with the formation of cross-linked polymers.^5e^ Even more recently, Cameron et al.^5f^ investigated the reaction of Cys with allenamides, following the work of Loh et al.,^5g^ but preparing modified cyclic peptides by intramolecular addition.

In this context, a general comparison of the adducts of representative thiols to a series of acceptors, which would not only include the maleimide ring and the activated double or triple bonds mentioned in the preceding paragraph, would shed light on the issue of the relative thermodynamic and kinetic stabilities of these adducts and on the search for alternative linkers.

**Scheme 1:**
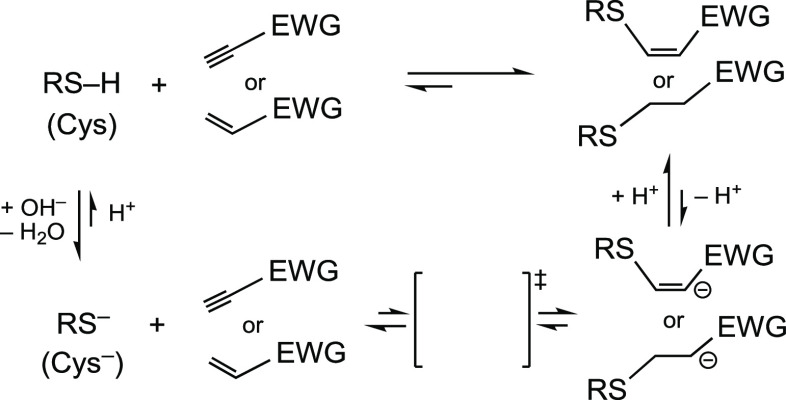
Addition of Thiols to Activated Triple and Double Bonds, Which in Aqueous Media at pH ≥ 6 Takes Place through Thiolate Ions

To this end, we first compared the equilibria shown in the upper row of Scheme 1, to evaluate the energies of the adducts in relation to their precursors, that is, the relative thermodynamic stabilities of these adducts, or the relative feasibility of the Michael addition with regard to the retro-Michael reaction.

From a kinetic point of view, these addition reactions take place:^2,3e,5b^ (a) under nucleophilic catalysis, where zwitterionic intermediates (generated in a first step from the acceptor and the catalyst) deprotonate RSH and the resulting RS^–^ ions attack on the electrophilic carbon atom(s); (b) under basic catalysis in organic solvents, with involvement of RSH···B species; and (c) in aqueous media close to neutral pH or, even more rapidly, at basic pH,^7^ that is, through the participation of thiolate ions as shown in the lower row of Scheme 1. Since standard bioconjugation reactions take place in water, we have focused our interest on this last case, that is, in the kinetics of the process in water for the various acceptors. Either trace amounts of aliphatic thiolates, as are usually present in neutral aqueous media, or significant amounts of thiolates, as it happens in basic aqueous media, are then involved. The true percentages of the thiolate anions will obviously depend on the p*K*_a_ of each sulfanyl/sulfhydryl/mercapto group.^7^ Since it is known that in aqueous media, activated triple bonds are mainly converted into *Z* adducts,^5b,6^ for the sake of simplicity, the *E* adducts (which are predominantly formed in organic solvents, in the presence of tertiary amines) are not included in Scheme 1 and in many of the following schemes and figures.

Apart from the thermodynamics and kinetics of the reactions mentioned in the preceding paragraphs, we would like (a) to gain insight into all the mechanistic details; (b) to confirm or discard explanations about why *Z* adducts are mainly obtained in aqueous media; (c) to study in silico the double addition of thiolates to activated triple bonds, which is a possible cause of instability of the *Z* adducts; and (d) to analyze the pros and cons of ethynesulfonamides, ethynesulfinamides, ethynephosphonamidates, and other HC≡C–EWG as alternatives to maleimides in bioconjugation reactions.

## Results and Discussion

### Thermodynamic Stability of the Adducts

**Scheme 2:**
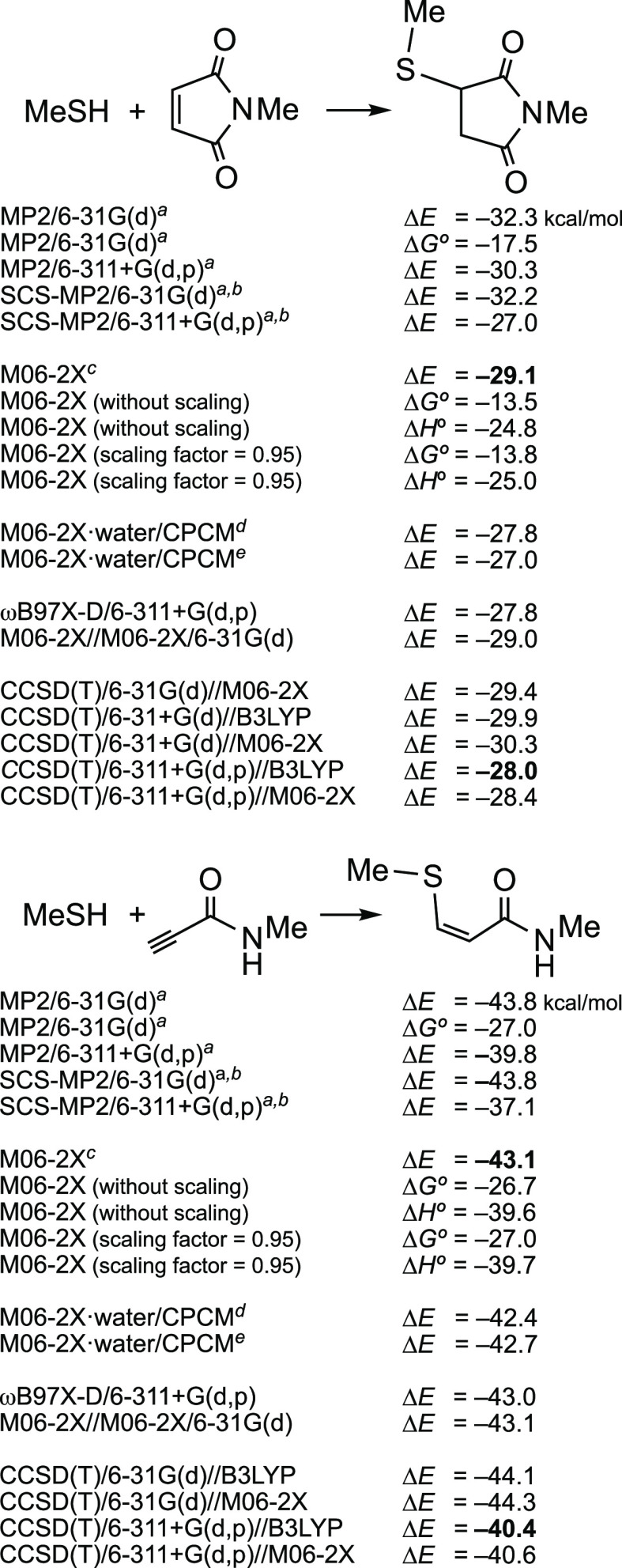
Reaction Energies Calculated for Two Reaction Models Single-point calculations from B3LYP/6-31G(d) geometries. Calculations with ORCA. M06-2X = M06-2X/6-311+G(d,p), throughout this work. Optimization in water with Spartan’18.2. Optimization in water with Gaussian 16.

With methanethiol (CH_3_SH/MeSH) as a model, the upper-row reaction of Scheme 1 was computed at several levels of theory for a series of acceptors. Two examples of results, for *N*-methylmaleimide and *N*-methylpropynamide, are shown in Scheme 2.

It is observed in Scheme 2 that the reaction of MeSH with *N*-methylmaleimide is predicted by all methods to be similarly exothermic (for example, Δ*E* ≈ −29 kcal/mol with M06-2X/6-311+G(d,p), henceforward M06-2X, and Δ*E* ≈ −28 kcal/mol at the highest level examined here). The addition of MeSH to *N*-methylpropynamide is even more so: around −43 kcal/mol with M06-2X and around −40 kcal/mol with CCSD(T)/6-311+G(d,p). For details and comparisons, see the Supporting Information. The estimated Δ*H*° values, obtained from frequency calculations, do not differ too much from the Δ*E* values, as expected. The estimated Δ*G*° values (Gibbs free energies, or free enthalpies), with and without scaling factors, are 16 ± 1 kcal/mol above Δ*E* values, which is a reasonable value of the *T*·Δ*S* term for addition reactions (two molecules being converted into one product). It states that both reactions are highly exergonic, around −15 kcal/mol in the first case and around −27 kcal/mol in the second case. These approximate numbers are sufficient in the present context. Henceforward, for the sake of simplicity and to save a lot of computer time, we will compare the total energies as obtained directly from the calculations, bearing in mind that we would have to add around 16 kcal/mol to the Δ*E* values to obtain approximate Δ*G°* values. Calculations in water (CPCM) did not change significantly the results (see the Supporting Information).

**Figure 1:**
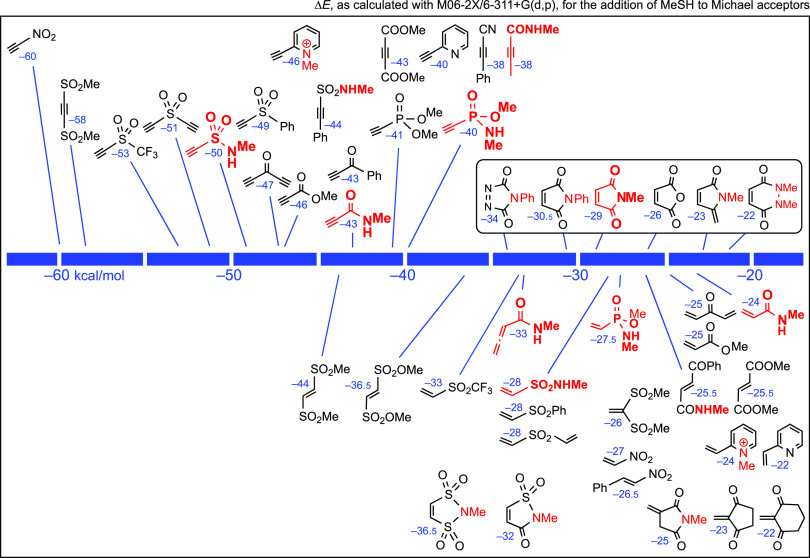
Relative stability, in kcal/mol, of the addition products of MeSH to known or potential acceptors.

We proceeded similarly with 45 additional reactions. The corresponding M06-2X-calculated reaction energies, from the lowest-energy conformer of each molecule, are shown in Figure 1. As indicated, we chose this method in all the figures, as a comparison tool. However, as mentioned above, methods such as those indicated in Scheme 2 were sometimes used with these additional reactions, to detect differences; in general, they afforded similar results to M06-2X. Analogously, the gaps between Δ*E* values and Δ*G°* values were around 16 ± 1 kcal/mol for several additional equilibria.

Those reactions that are more exothermic are located on the left in Figure 1. Thus, triple bonds linked to the strongest EWGs, such as NO_2_ and SO_2_CF_3_, are predicted to afford the relatively more stable adducts, MeS–CH=CH–EWG, where the resonance energy of the system can explain this. In contrast, the additions of MeSH to activated double bonds, to afford MeSCH_2_CH_2_EWG, appear on the right in Figure 1; they are much less exothermic. These results are not surprising, as it has been known since the beginnings of organic chemistry that triple bonds have a higher propensity to react with nucleophiles than double bonds. In this case, Figure 1 predicts the relative thermodynamic stability of each adduct in relation to its precursors.

Michael acceptors that are amides or imides (maleimide and relatives) are highlighted in red in Figure 1, to indicate that the spacers would be covalently bound to the corresponding N atoms or to carbon atoms linked to these N atoms. In other words, functionalized long chains would appear there in lieu of Me groups, in practice. Substrates with NPh groups are representatives of real linkers functionalized at the C4 (or C3) position of Ph, where once again the spacers would be bound. *N*-Phenyl derivatives were only occasionally included in Figure 1, for the sake of simplification. A phenyl group produced a small shift to the left, in relation to a methyl group. For example, for HC≡C–CONHPh and HC≡C–PO(OMe)NHPh, the Δ*E* values were –44 and –42 kcal/mol, respectively.

Figure 1 also suggests that pyridinium cations stabilize the adduct of MeSH to unsaturated bonds more than the neutral pyridine, as experimentally observed,^5d^ but not to a great extent (Δ*E* = −46 vs −40 kcal/mol for a triple bond, −24 vs −22 kcal/mol for a double bond). Figure 1 also shows that the allenamide,^5f,5g^ is a better acceptor (Δ*E* = −33 kcal/mol) than the analogous propenamide (acrylamide, −24 kcal/mol), but it is less than its related propynamide (−43 kcal/mol) and 2-butynamide (−38 kcal/mol). We can state that the allenyl group is “intermediate” between ethynyl (acetylenyl) and ethenyl (vinyl) groups.

Finally, since we were particularly interested in comparing different types of amides with maleimide and with methyl propynoate, which we took as reference compounds, an excerpt of Figure 1 follows:
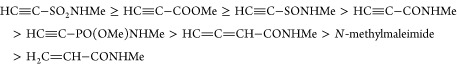
In this order of stability, we have added *N*-methylethynesulfinamide, for which Δ*E* = −46.5 kcal/mol, for the sake of comparison. However, we did not include sulfinamides in Figure 1 because we were not interested in using chiral compounds (see below).

Most of the reactions included in Figure 1 were recalculated in the presence of polar solvents, with implicit-solvent models, mainly in water, as we did in Scheme 2. The changes in relation to the calculations for isolated molecules (gas phase, under vacuum) were small: ±2 kcal/mol. In other words, the polarity of the solvent was predicted to be insignificant in these equilibria that only involve neutral molecules.

### The Cases of AcCysOMe and *^t^*BuSH

It may be argued that Cys-derived adducts would not be as stable as MeSH adducts, bearing in mind the EWG effect due to the presence of polar groups in a protein chain. The total energies of the reactions of methyl *N*-acetylcysteinate (AcCysOMe) with *N*-methylmaleimide, *N*-methylpropynamide, and (*N*-methyl)ethynesulfonamide were optimized at the M06-2X level, in the gas phase (Gaussian 16) and with water as the implicit solvent (CPCM, Spartan’18.2). As always, only the lowest-energy conformers of each species are depicted. More details are given in the Supporting Information.

**Scheme 3:**
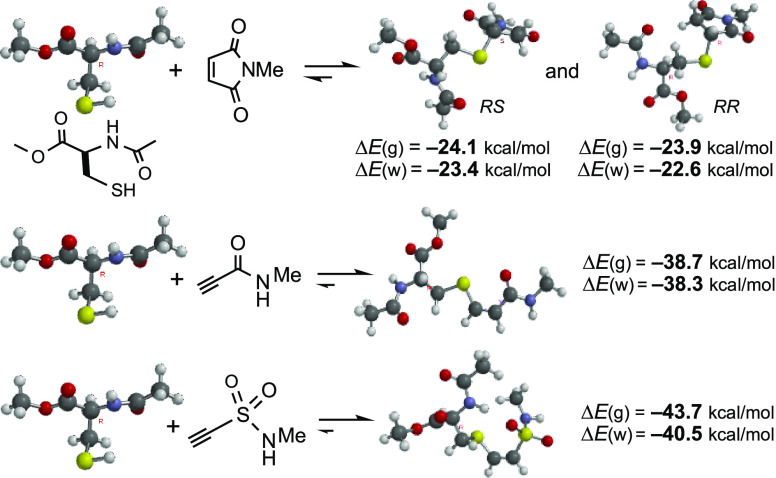
Energies Calculated for the Reactions of AcCysOMe with Some Activated Double or Triple Bonds, by Means of the M06-2X/6-311+G(d,p) Method

In the case of maleimide, both diastereoisomers, *RS* and *RR*, were examined: the difference, see Scheme 3, was only of 0.2 kcal/mol (0.8 kcal/mol in water/CPCM). The fact is that mixtures are obtained in the additions of chiral thiols to maleimides.^5b^

As shown in Scheme 3, the adducts from triple bonds are relatively more stable than those from maleimide, as in Figure 1. Since sulfonamido groups are stronger EWGs than their carboxamido counterparts, it is reasonable to observe that the third equation is even more shifted to the right than the second. Comparison of the reactions in Scheme 3 with those in Scheme 2 and Figure 1 indicates that those involving AcCysOMe are 5 ± 1 kcal/mol less exothermic than those with MeSH, which reflects the electron-withdrawing features of the Cys functional groups, making the S–C bond weaker. To save calculation time, we continued using MeSH as the model, although a correction of around 5 kcal/mol may be necessary, in general.

We also calculated the case of *^t^*BuSH (1,1-dimethylethanethiol) to check the effect of a large alkyl group (the possible steric hindrance): Δ*E*(g) = −27.0 and Δ*E*(w) = −25.1 kcal/mol for the addition to *N*-methylmaleimide; Δ*E*(g) = −42.0 and Δ*E*(w) = −41.0 kcal/mol for the addition to *N*-methylpropynamide; and Δ*E*(g) = −47.8 and Δ*E*(w) = −45.9 kcal/mol for the sulfonamide. A difference of ∼2 kcal/mol is thus noted between the *^t^*BuS and MeS adducts.

### Anionic Intermediates: Kinetics

The addition of aliphatic thiols to Michael acceptors is very slow in acidic aqueous media—pH values > 6.0 are usually required for a rapid reaction^3c,5b^—but in slightly basic media, the concentration of the thiolate anions increases and the reactions are then extremely rapid with most acceptors. Obviously, the reactivity depends both on the acidity of the thiol, the nucleophilicity of the corresponding anion, and the electrophilicity of the Michael acceptor. Anyway, the difference of nucleophilicity between thiolate ions and neutral thiols is spectacular, as known and in accordance with the calculated HOMO energy for MeS^–^ of −0.75 eV and that for MeSH of −8.02 eV, both at the M06-2X level.

**Figure 2:**
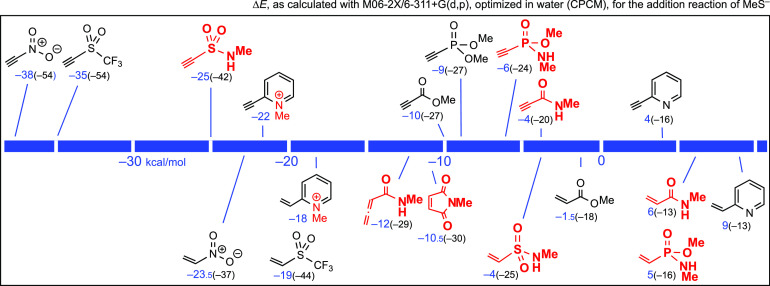
Total energies in kcal/mol for the addition of MeS**^–^** to representative acceptors in water. Within parentheses, in the gas phase.

Figure 2 shows the total energies of the reactions of MeS^–^ with a selection of electrophilic triple or double bonds,^8^ to afford the corresponding anionic adducts, with water as the implicit solvent (energies highlighted in blue), and in the gas phase (values within parentheses). Since the bioconjugation of Cys takes place in aqueous media, we give more importance to the results obtained in water. We examined different models, especially the CPCM method implemented in Spartan’18.2 and in Gaussian 16, with similar results (see Computational Methods and the Supporting Information). Now, the effect of water is spectacular, as it could be expected bearing in mind that anionic species are involved.

Again, the activated triple bonds are on the left side in Figure 2. However, the activated double bonds are relatively shifted to the left in Figure 2, in relation to Figure 1. Now, *N*-methylmaleimide surpasses the propynamide and the ethynephosphonamidate. This has an obvious explanation: the stabilization of sp^2^ or vinyl anions (by resonance with allenolate-like canonical forms) is less than that of sp^3^ anions (by resonance with enolate-like canonical forms).

For the pyridinium derivatives, the difference between the ethynyl- and vinyl-substituted substrates was also reduced (see Figure 2). In other words, with methanethiolate ions in aqueous medium, the calculations predict that the key step of each overall process—the addition of the anion to the substrates—is almost equally shifted to the right in both cases. It is also worth noting that the addition of MeS^–^ to 2-ethynyl-*N*-methylpyridinium ions is as favorable as that to *N*-methylethynesulfonamide; nevertheless, if a counterion such as BF_4_^–^ is added in the calculations of the ethynylpyridinium ion, the predicted reaction energy in water is −18.2 instead of −22 (−21.9) kcal/mol, while that for the vinylpyridinium ion is −15.4 instead of −18.0 kcal/mol.

It is interesting to note the allenic carboxamide^5f^ (*N*-methyl-2,3-butadienamide) at the left of *N*-methylmaleimide. The delocalization of the negative charge in the anionic adduct can explain this result.

Since the term *T*·Δ*S* may be estimated to be around 16 ± 1 kcal/mol (see Scheme 2), a value that should be added to the numbers disclosed in Figure 2, it seems that in water and aqueous solvents, the equilibria are not shifted toward the anionic adducts for the Michael acceptors that are on the right in Figure 2. The protonation of these anionic adducts is required to complete the addition. By contrast, in the gas phase and presumably in nonpolar solvents, almost all the additions are predicted to be largely exothermic. Since most reactions that are interesting in the present context involve physiological conditions, we will focus our attention on the results obtained in water or in water–polar solvent mixtures.

**Figure 3:**
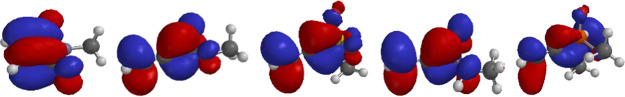
M06-2X-predicted LUMOs for the lowest-energy conformers of *N*-methylmaleimide (−1.84 eV), methyl propynoate (−0.43 eV), (*N*-methyl)ethynesulfonamide (−0.02 eV), *N*-methylpropynamide (−0.02 eV), and methyl (*N*-methyl)ethynephosphonamidate (0.33 eV).

Comparison of the LUMO energies of the lowest-energy conformers of *N*-methylmaleimide, methyl propynoate, (*N*-methyl)ethynesulfonamide, *N*-methylpropynamide, and methyl (*N*-methyl)ethynephosphonamidate in the gas phase (Figure 3) suggested that the reactivity of these Michael acceptors with nucleophile reactions may approximately follow this order. Two relatively close π_z_ and π_y_ LUMOs were found for the sulfonamide (−0.02 and 0.09 eV) and the phosphonamidate (0.33 and 0.45 eV) due to the special features of triple bonds attached to tetrahedral S and P atoms. The order is similar with water as the implicit solvent.

Going farther, M06-2X calculations of the corresponding transition states (TSs),^9^ with MeS^–^ in water, indicated that the barriers, given below in kcal/mol, are very low:
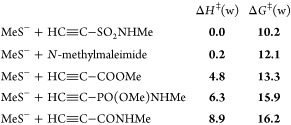


These values qualitatively agree with the values for the intermediates subsequent to these TSs that are shown in Figure 2. In short, these additions are predicted to be extremely quick even in water, as “click” reactions, especially the first two: sulfonamides due to the strong EW character of the SO_2_ group; maleimides due to their π electron system (of enediones, with the LUMO energies lower than those of simple enones). In other words, the reaction rates of this key step in water are expected to follow the approximate order:
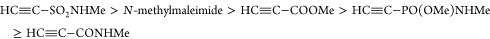
Figure 2, from left to right, provides an approximate ordering of the reaction rates for the set of compounds studied. In a first approach, the higher the stabilization of the negative charge of the anionic intermediates, the higher the reaction rates of the Michael addition.^10^

### Mechanistic Comparisons

**Scheme 4:**
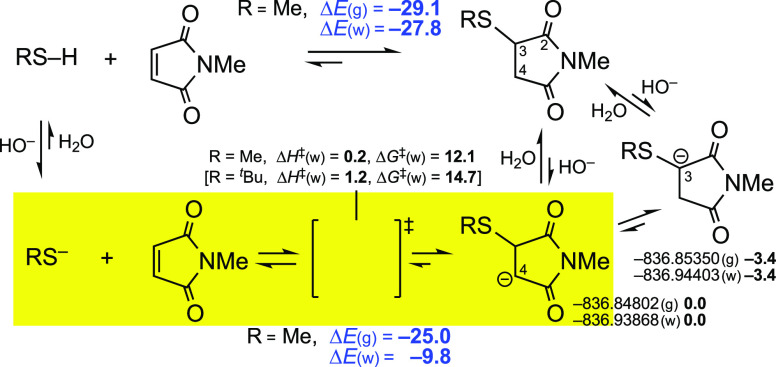
Steps Involved in the Addition of Thiols to Maleimides Δ*E*, Δ*H*, and Δ*G* values in kcal/mol.

Once the energies for the overall reactions and for the additions of the anions had been calculated, we compared the plausible intermediates for the selected cases of maleimide, propynamide, ethynephosphonamidates, and ethynesulfonamides. Scheme 4 reviews the steps mentioned above for the addition of methanethiol, via its anion, to maleimides, but also the TS for the addition of *^t^*BuS^–^, and shows a detail worthy of mention: the initial anion from the conjugate addition (the delocalized enolate-type anion, at C4) can easily be isomerized to the delocalized or enolate-like anion at C3, which is ∼3 kcal/mol more stable, by the effect of the S atom.

Experimentally, we had observed^5b^ that, when the reaction of methyl *N*-acetylcysteinate (AcCysOMe) and *N*-benzylmaleimide [*N*-(phenylmethyl)maleimide] was carried out in D_2_O (with a small amount of K_3_PO_4_), the adduct (**1**) was deuterated at both C3 and C4 of the succinimide ring. Deuteration at C4 is a consequence of the expected *anti*-addition of RS^–^ and D^+^. Deuteration at C3 is spontaneous in slightly basic media, as H3 is a relatively acidic proton.

**Scheme 5:**
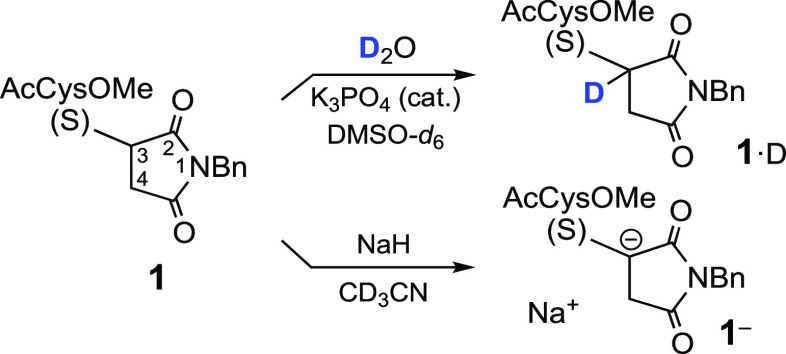
Summary of Experiments Followed by NMR Spectroscopy

Moreover, we have corroborated that the simple dissolution of adduct **1** in D_2_O/DMSO-*d*_6_, in the presence of trace K_3_PO_4_, affords a C-monodeuterated compound. The ^1^H and ^13^C NMR spectra clearly indicate that the methine proton close to S has been replaced by D (see **1**·D in Scheme 5). As just mentioned, the methine protons (H3) of these succinimide derivatives are likely more acidic than the neighboring methylene protons (H4a/H4b). Thus, the calculations (Scheme 4) agree with the available experimental data. In another experiment, the addition of a stronger base (NaH) in CD_3_CN results in the almost full disappearance of H3 (but in this case also the AcNH signals of the two diastereomers and H4 partially decreased).

**Scheme 6:**
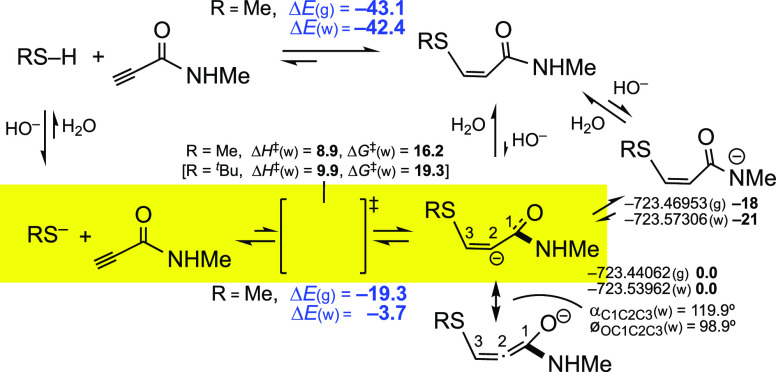
Steps Involved in the Addition of Thiols to Propynamides Δ*E*, Δ*H*, and Δ*G* values in bold, in kcal/mol.

Scheme 6 reviews the steps mentioned above for the addition of MeSH, via its anion, to a propynamide, but also the TS for the addition of *^t^*BuS^–^. It includes the intramolecular prototropy that may occur during the addition of MeSH/MeS^–^ to *N*-methylpropynamide, as the first anionic intermediate (allenolate-type, a hybrid structure of the two canonical forms shown in Scheme 6, with a predicted C1–C2–C3 angle of 123.7° in vacuo and 119.9° in water) can be converted into an enamide anion, which is 18–21 kcal/mol more stable.^11^

A similar prototropy can occur in the cases of the corresponding anionic adducts of phosphonamidates and sulfonamides, but the energy gain is lower [Δ*E*(g) = −9.1 and Δ*E*(w) −12.2 kcal/mol for the phosphonamidate case, and Δ*E*(g) = −7.9 and Δ*E*(w) −12.9 kcal/mol for the sulfonamide case] than in Scheme 6. This must be due to the nature of carboxamido groups, that is, to their paradigmatic stabilization by resonance, which is stronger than for phosphonamidates and sulfonamides, with P–N and S–N bonds longer than C(O)–N bonds.

**Scheme 7:**
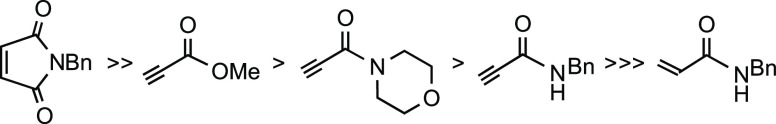
Experimental Order of Reactivity for Representative Cases

In aqueous media, the protonation of the first anionic intermediate is expected to be instantaneous, so it is likely that these amide anions do not play any practical role in bioconjugation reactions. Kinetically, only the true concentration of the thiolate ions (that is, the pH of the medium) and the barriers for the addition of these thiolate ions to the Michael acceptors are crucial. Experimentally,^5b^ we had observed that at pH 7.4, in H_2_O–*^t^*BuOH to ensure that all the starting compounds remained soluble, the order of reaction rates of AcCysOMe with several acceptors was that reproduced in Scheme 7. Rather than determining and ensuring the rate constants at pH 7.4 under pseudo-first-order conditions,^5b^ we have now compared the relative reactivity of AcCysOMe under standard laboratory conditions (0.1 M, rt, equimolar amounts of reactants), in buffer pH 6.0 plus *^t^*BuOH (1:1 v/v). The reactivity order (Scheme 7) is maintained: with the maleimide, the reaction was complete within 30 min; methyl propynoate required 1 h; the morpholine propynamide required overnight stirring; the reaction of *N*-benzylpropynamide was complete within 2 days; and *N*-benzylpropenamide did not react at all after 2 days (only 10% conversion after 2 days at 50 °C).

**Figure 4:**
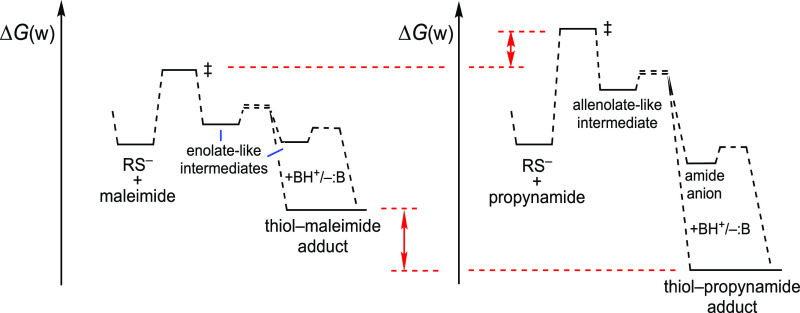
Estimated reaction profiles for the addition of thiolates to *N*-methylmaleimide and to *N*-methylpropynamide.

To summarize, when comparing the reaction profiles of maleimides with propynamides, it turns out that C(sp^3^) carbanions that can delocalize the charge on a neighboring CO group are more stable than the at-first-sight intrinsically favored C(sp^2^) carbanions, since the stabilization of the latter by resonance is lower. In other words, as Figure 4 shows, the step from maleimide to the first anionic intermediate is kinetically favored with respect to the higher-barrier step from the propynamide to MeSCH=C^–^CONHMe, the alkylsulfanylpropenamide anionic intermediate, but the thiol–maleimide adduct is thermodynamically less stable than the thiol–propynamide adduct (and than the thiol–sulfonamide and thiol–phosphonamidate adducts).

### Why Are *Z* Adducts Mainly Obtained in Aqueous Media?

As mentioned in the Introduction, mixtures of *Z* and *E* adducts are expected when thiols add to activated triple bonds (calculations indicate that *E* adducts are usually around 1 kcal/mol more stable than their *Z* counterparts). However, in aqueous media this is not the case, as *Z* adducts largely predominate.^5,6^ Calculations of the corresponding intermediates would allow us to gain more insight into this issue.

As already shown in Scheme 6, the anionic intermediates arising from the addition of MeS^–^ to *N*-methylpropynamide can be viewed as hybrid structures between two canonical forms (vinyl anion and allenolate anion) with NHR′ substituents at C1. Furthermore, they may be in equilibrium with their configurational isomers (*E*), likely through these allenolate-like species, or through the corresponding allenol-type intermediates (if the O atom is protonated, by intramolecular proton migration).

**Scheme 8:**
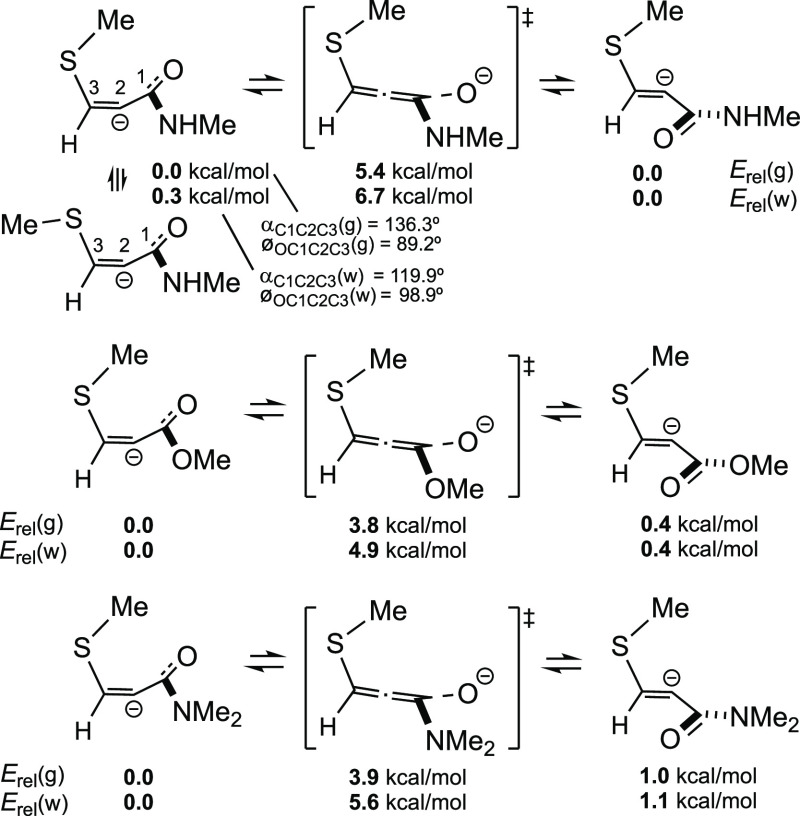
Relative Stability of Initial Allenolate-Type Intermediates Generated from MeS^–^ and Some Activated Triple Bonds and Their Possible Isomerization

Scheme 8 indicates that these special *E*-type anions have similar energies than the anions of their associated *Z* isomers and that the inconversion barriers are very low. Equilibration of the anions must therefore be very rapid. If it is not produced in aqueous media, it must be due to the even more rapid protonation of the initially formed *Z*-like anion, as it is generally accepted.^6^

Finally, Scheme 8 shows that, for the anions, the MeS-folded conformers, with the Me group over the delocalized anionic charge, have lower energies than those with the *ap* MeS rotamer (Me antiperiplanar to C2, or Me–S–C3–C2 dihedral angle ≈ 180°), although there is an exception for the first example in water. Nevertheless, the differences between these rotamers are generally ≤1 kcal/mol, so the use of one or another for the calculations of reaction energies, which are usually very large numbers, is not relevant.

### Double Addition of Thiolates to Activated Triple Bonds: Exchange of Thiolates via Double Addition

The conjugate addition of one molecule of RSH to one molecule containing an activated triple bond (electrophilic alkyne) gives rise to a product that is still unsaturated. Although the steric and electronic effects of the RS group are expected to decrease the electrophilicity of the β carbon atom, a second addition is still feasible. This is well known.^1−3,12^ However, the double addition is contraindicated if a controlled or relatively homogeneous drug-to-antibody ratio (DAR) is wanted, in the field of antitumor ADCs. The chemical and enzymatic stability of the covalent bonds of ADCs while circulating in the blood is vital.

**Scheme 9:**
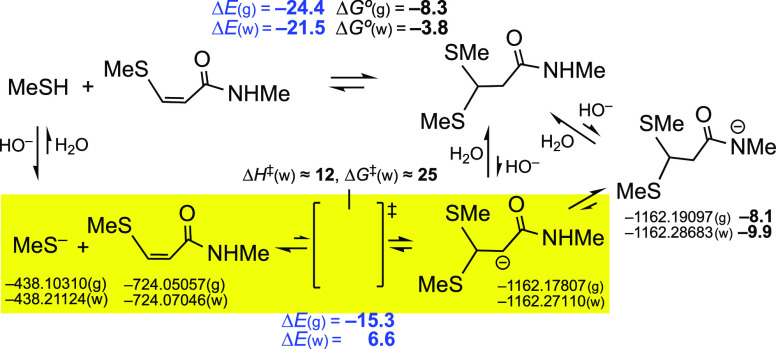
Analysis of the Double Addition of MeSH to *N*-Methylpropynamide Relative gaps and reaction energies in kcal/mol.

It is known that the second addition of thiols to some alkynones is up to 1000 times slower than the first addition.^12a^ However, many dithioacetals of the (RS)_2_CHCH_2_COR/Ar type have been isolated.^5e,13^ Particularly, we did not observe a double addition to propynamides under physiological conditions,^5b^ but it does not exclude that thiol exchanges may occur by heating in suitable non-polar solvents. Anyway, we were interested in explaining why some double additions are less probable than others, by means of DFT calculations. First, Scheme 9 discloses our results, at the M06-2X level [M06-2X/6-311+G(d,p)] as always, with the main model compounds—MeSH and *N*-methylpropynamide—used throughout this work.

Kinetically (Scheme 9, bottom equation), the attack of MeS^–^ in water has a high barrier [Δ*G*^⧧^(w) = 25 kcal/mol] and the anionic intermediate is 6.6 kcal/mol [Δ*G°*(w) ≈ 22 kcal/mol] above its precursors: the addition is expected to be negligible in physiological media. Thermodynamically, however, the values of −24.4 kcal/mol in vacuo and –21.5 kcal/mol in water for the top equation in Scheme 9, for which the calculated values of Δ*G*° were –8.3 and –3.8 kcal/mol, respectively, appear to indicate that the reaction is still feasible, but this is a very simple case. With large thiols (*^t^*BuSH), the corresponding reactions with the anions—kinetics—were calculated to be around 4 kcal/mol more endothermic and endergonic and with the neutral thiols—thermodynamics—to be also less favorable. It is expected that with Cys-containing peptides or proteins, the corresponding double additions would be even less possible, kinetically and thermodynamically.

**Scheme 10:**
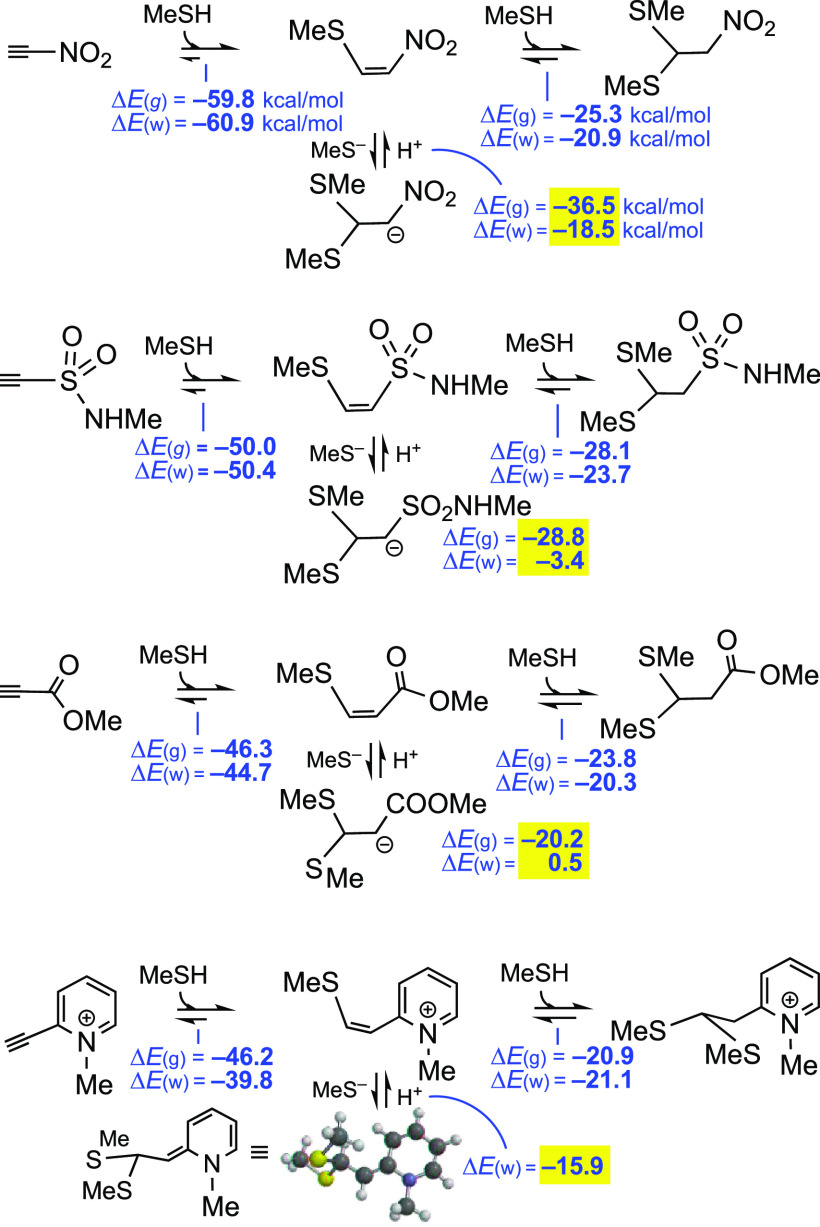
Comparison of the Mono and Double Addition of MeSH and of MeS^–^ to Representative Triple Bonds

Finally, in Scheme 10, the energetic differences between the first and second addition for four representative cases are compared to the propynamide case already shown in Scheme 9. First of all, the M06-2X method indicates that the double addition is ≥20 kcal/mol less shifted to the right than the first addition. In other words, the second addition of MeSH is thermodynamically less favorable than the first addition, as expected and in agreement with the chemical literature.^5,6,12,13^ It is also predicted, to our initial surprise, that the double additions of MeSH to the different acceptors have quite similar Δ*E* values, especially in water. Thermodynamically, these further examples are thus predicted to be quite favorable, at least in the gas phase and, probably, in nonpolar solvents, as has been experimentally demonstrated for ynones.^5e^

Kinetically, the comparison may be established via the addition of MeS^–^, as often done in this work, that is, by the relative stability of the anionic intermediates, bearing in mind that the same factors that lower the energy of the anionic intermediates would lower the energy of the preceding anionic TSs (Hammond’s postulate, in simple terms), saving time by avoiding the characterization of all the TSs. These results are also included in Scheme 10 (vertical chemical equations).

It is observed that the strongest EWGs afford the more negative values of Δ*E* for the formation of these anions (double addition, as qualitatively expected at first sight, but the calculation results allow for more reliable comparisons. The second addition of MeS^–^ in the gas phase, and presumably in nonpolar solvents, is kinetically favored for almost all groups, particularly for the *N*-methylpyridinium ion (where the energy due to the coupling of a cation and an anion is a huge number), NO_2_, and SO_2_NHMe groups. In water, in view of Schemes 9 and 10, the order of relative stability is predicted to be
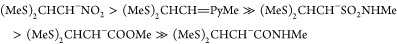


This list may be extended with all the triple bonds shown in Figures 1 and 2, but it seems unnecessary: the stronger the electron-withdrawing character of the substituent, the higher the stabilization of the negatively charged intermediate and hence the higher the reaction rate of the second addition. All in all, it appears that the differences between the Michael acceptors shown in Schemes 9 and 10 are mainly of a kinetic origin and that those good Michael acceptors on the left in Figure 2 can also show a parallel chance of double addition.

The second addition of a nucleophile to the triple bond has potential drawbacks, as is known.^5,6^ First, if the anionic intermediate, (MeS)_2_CHCH^–^EWG, is formed, even in minute amounts, the partial isomerization of *Z* to *E* adducts can occur via this pathway, a standard addition–elimination (AE) mechanism. Second, an exchange of thiol groups may occur via the double adduct. This cause of instability is different from that of maleimides—hydrolysis of the succinimide ring, retro-Michael reaction—but it is also undesired.

### Pros and Cons of Ethynesulfonamides, Ethynesulfinamides, and Ethynephosphonamidates

Sulfonamides are the strongest Michael acceptors among the amino-containing compounds examined here. Kinetically they should be the linkers of choice, but their reactivity may be a handicap for their chemoselectivity, as they can undergo addition of N-nucleophiles and O-nucleophiles, including water, much weaker than RS^–^. Also, acetylenic sulfones, alkyl ethynesulfonates, and ethynesulfonamides are much more prone to polymerization than alkyl propynoates and propynamides, as we are aware.^6g^ This also occurs with maleimides, which are very prone to anionic and radical polymerizations.^14^ The preparation and storage of HC≡C–SO_2_NHR and their precursors, HC≡C–SO_2_–LG, are not as simple as that of propynamides and their precursors. Additionally, the tendency of ethynesulfonamides to undergo double addition of thiol groups, mentioned in the preceding section, although interesting from the viewpoint of the dynamic combinatorial chemistry,^5e,6d,6e^ was disappointing for us.

Sulfinamides and phosphonamidates are chiral compounds, so that the attack of natural Cys (L, *R*) or any Cys-containing protein on the corresponding ethynesulfinamide (acetylenesulfinamide) and ethynephosphonamidate will give rise to diastereomeric mixtures (*RR* and *RS*). If the objective is the preparation of an ADC with a DAR of 4 ± 1, formation of mixtures of stereoisomers does not matter: what is important is that the ADC arrives intact at the destination and destroys or kills the tumor cells. However, if the linker is planned for the isotopic labeling or fluorescence tagging of a peptide with some Cys units or residues that has to be purified and characterized spectroscopically, sulfinamides and phosphonamidates should be ruled out. Phosphonodiamides could be used instead of phosphonamidates, but the value of Δ*E* = −37 for the *N,N*-dimethyl derivative, not included in Figure 1 for the sake of simplification, suggests that they are not a promising option.

Therefore, a compromise choice is required between the acidity of the thiol (since under physiological conditions at least some quantities of RS^–^ must be present for a rapid reaction), the reaction rate of the key addition step, the subsequent equilibrium steps, the stability of the final adduct, and the possible involvement of this final adduct in other reactions, such as retro-Michael reactions on heating, double additions with subsequent thiol exchanges, or *Z*-to-*E* isomerizations via allenolate-type intermediates or via double addition.

## Conclusions

According to Figure 1, the general rule is that triple bonds linked to sulfonyl groups, pyridinium cations, carbonyl groups, carboxyl groups, carboxamido groups, phosphonic esters, and phosphonamidates afford thermodynamically more stable thia-Michael adducts (with respect to the precursors) than heterocyclic enediones such as *N*-phenylmaleimide, *N*-methylmaleimide, and maleic anhydride, or than other activated double bonds. The resonance between S, CH=CH, and EWG explains the extra thermodynamic stability of the adducts arising from activated triple bonds. Of course, calculations “only” allow one to evaluate how large is this relative stabilization for a manifold of low-energy conformers for each configurational isomer or chemical entity. Many acceptors have been briefly discussed, to save space and/or because of our goal of linking Cys-containing proteins with amino-decorated drugs, which focused our attention on the different types of amides, but readers interested in other substrates may reach their own conclusions from Figure 1. Application of these results to the hot topic of covalent binding, particularly to thiol-binding drugs,^15^ is quite straightforward but it is outside the scope of the present work.

Figure 2, in contrast, provides an idea of the reaction kinetics. In Figure 2, the energies of the reactions of Michael acceptors with equimolar amounts of thiolate ions (instead of thiols) have been estimated. In basic aqueous media, 2-vinylpyridinium salts, *N*-methyl-2,3-butadienamide, *N*-methylmaleimide, and other double bond-containing acceptors gain positions in the “ranking”. The new order, with the electrophilic double bonds shifted to the left in Figure 2, with regard to Figure 1, has an obvious reasonable explanation: the delocalization of the negative charge, in the anionic adduct, on the neighboring CO group or EWGs in general. The stabilization by resonance due to an allenolate-like ion is not so large.

Therefore, the compounds on the left with respect to maleimides in Figures 1 and 2 are predicted to be the best alternatives when thiolate ions can be involved. In particular, the ideal acceptors for bioconjugation have appeared to be ethynesulfonamides, because they are the “best” in terms of our figures, from thermodynamic and kinetic points of view: the calculations predict that they combine the highest stability of the thiol adducts with the highest reaction rates with thiolate ions. The problem is practical. It is key to avoid excessive reactivity, including the tendency of reagents HC≡C–SO_2_–LG to polymerize, in the presence of anionic or radical initiators. It is also crucial to bypass the tendency of Cys(S)–CH=CH–SO_2_NH–spacer–drug adducts to undergo double addition, with possible exchanges and with partial *Z*-to-*E* isomerizations via these double addition intermediates. For the moment, we have focused mainly on the development of linkers arising from yne-carboxamides as a compromise choice. This is so because their stability and ease of preparation, despite the fact that the reaction rates of the addition step, at identical concentration of thiolate ions, are not so high as those of yne-sulfonamides and maleimides. However, we have not ruled out studying suitable non-terminal yne-sulfonamide derivatives in the future that are less reactive and more selective than HC≡C–SO_2_NHR.

From a more general point of view, the simple addition of thiols to activated triple bonds may be deemed a classical conjugate addition, with features of a click reaction. However, to our initial surprise, once examined in depth, it turned out to be really complex in terms of kinetics, configuration of the adducts, plausible exchange reactions, and double addition or configuration change via such a double addition. We have shed further light, mainly by means of the M06-2X/6-311+G(d,p) DFT method, as well as with some NMR experiments, on these complex issues.

## Experimental Section

### Computational Methods

The Gaussian 16 package was always used,^16^ but in some cases, as indicated, some calculations were also repeated with the Spartan’18.2^17^ or ORCA^18^ software. The total energies, *E*, are in au or Hartrees; the differences, Δ*E*, are given in kcal/mol (1 au = 627.5 kcal/mol). The M06-2X/6-311+G(d,p) method,^19^ often abbreviated as M06-2X to gain space in schemes and figures, was used throughout. All the discussions are based on the results provided by this method; for more details, see the Supporting Information. In addition, the low-cost MP2/6-31G(d)//B3LYP/6-31G(d) approach, which does not overestimate the London dispersion forces as much as MP2/6-311+G(d,p),^20^ was initially employed to choose the lowest energy conformer(s) for the species with a huge number of possible rotamers. For simplicity and to gain space, we refer these MP2/6-31G(d)//B3LYP/6-31G(d) calculations as MP2. Other methods—either intermediate-level DFT, the spin-component scaled MP2 (SCS-MP2),^21^ or correlated wave-function methods such as CCSD(T), as shown in Scheme 2, but also see the Supporting Information for more details—were sometimes applied, to confirm the energy differences. This comparison was also useful to check their relative performance regarding the addition reactions under scrutiny; for example, as no difference was observed between the M06-2X/6-311+G(d,p) and M06-2X/6-311+G(d,p)//M06-2X/6-31G(d) energies, for substrates with a number of conformations larger than those in Scheme 2, we chose the “best conformers” using the minimal basis set and afterward we calculated the M06-2X/6-311+G(d,p) energies. From the frequency calculations with M06-2X/6-311+G(d,p), without corrections, *G* values were obtained, once it had been proven that a scaling factor of 0.95 (an assumed mean value from other estimated values for M06-2X with different basis sets, see the Supporting Information) does not change significantly the reaction energies, as shown in Scheme 2; the correction for B3LYP/6-31G(d) (see the Supporting Information) was added to the MP2 results. Transition states (only one imaginary frequency) were located at the M06-2X/6-311+G level, often after previous searches with lower-level DFT methods. Orbital and molecule drawings were obtained from Spartan’18.

The effect of water and other polar solvents on the reaction energies was estimated by optimization with several implicit-solvent models (CPCM, SMD, SSVPE, SM8) included in Gaussian 16 and/or in Spartan’18 packages (see the Supporting Information). The performance of these models is a hot topic under debate,^22^ with criticisms often addressed to the SMD and related methods when charged species are involved.^22^ As indicated in the corresponding schemes and figures, in the present work, the comparisons were mostly carried out by means of the CPCM method as implemented in Spartan’18 and in Gaussian 16; the results were very close, even though the total energy values were not identical (see the Supporting Information). The values of the total energies for the addition reactions may be approximate, but we were interested in the relative values, which turned out to be reasonable and in qualitative agreement with the currently available experimental values.

### NMR Spectra

Several reactions (see the Supporting Information) were followed by NMR spectroscopy. ^1^H NMR spectra were recorded on 400 MHz spectrometers and reproduced in the Supporting Information, with the solvent resonance as the internal standard (residual CHCl_3_ in CDCl_3_, 7.26 ppm; residual CD_2_HSOCD_3_ in DMSO-*d*_6_, 2.50 ppm; residual CD_2_HCN in CD_3_CN, 1.96 ppm). ^13^C NMR spectra were recorded at 100.6 MHz with proton decoupling; δ values are in ppm with respect to the solvent (CDCl_3_, 77.2 ppm; DMSO-*d*_6_, 39.5 ppm; CD_3_CN, 1.8/118.3 ppm).

## Supporting Information Available

Computational methods; complementary results; full Schemes of the main text; relevant M06-2X/6–311 + G(d ,p) equilibrium geometries; experimental; and exchange of thiolates in the maleimide adducts (PDF).
