# Supplementary material for: Computational Study of the Addition of Methanethiol to 40+ Michael Acceptors as a Model for the Bioconjugation of Cysteines
Source: J Org Chem. 2021 Apr 29;86(10):7107–18. doi: 10.1021/acs.joc.1c00349 (PMC8631706; doi:10.1021/acs.joc.1c00349)
Supplement: Supplementary file 1 — jo1c00349_si_001.pdf [file jo1c00349_si_001.pdf]

Supporting Information for

Computational Study of the Addition of Methanethiol to 40+ Michael Acceptors as Model for the Bioconjugation of Cysteines

Anna M. Costa,* Lluís Bosch, Elena Petit, and Jaume Vilarrasa*

Organic Chemistry Section, Facultat de Química, Universitat de Barcelona, Diagonal 645, 08028 Barcelona, Catalonia, Spain

Computational methods (additional details) SI-2

Complementary results SI-2

Full Schemes of the main text SI-3

Relevant M06-2X/6-311+G(d,p) equilibrium geometries (Cartesian coordinates) SI-7

Experimental

NMR spectra of **1**·D SI-10

Treatment of **1** with NaH SI-11

Migration of AcCysOMe from **1** to **2** SI-12

Exchange of thiolates in the maleimide adducts (mechanisms) SI-13

References SI-14

Computational methods (additional details)

All the calculations were carried out with the Gaussian 16 package.[S1] Some were repeated with Spartan'18 or with ORCA.[S2] The M06-2X/6-311+G(d,p) method was used everywhere.[S3] Many structures were first calculated at the MP2/6-31G(d)//-B3LYP/6-31G(d) level, which in our hands[S4] is the approach that afforded us the lowest performance/cost ratio for the appropriate ordering of sets of conformers and which we mention as MP2 or MP2/6-31G(d). Thus, comparisons were often established between MP2/6-31G(d) and M06-2X/6-311+G(d,p) total energies of the different molecules and anions; in other words, the several possible or reasonable conformers of every species were calculated and compared at these two levels. For large molecules, with many low-energy conformations, we confirmed that there were no significant differences (around 0.0001–0.0003 au, less than 0.2 kcal/mol) between M06-2X/6-311+G(d,p) and M06-2X/6-311+G(d,p)//M06-2X/6-31G(d), so that sometimes we took advantage of this approach for saving computing time.

The scaling factors used in Scheme 2 for the estimation of ΔH° and ΔG° from the frequency calculations are those published[S5] for several M06-2X methods [but not for M06-2X/6-311+G(d,p), so a mean value of 0.95 was taken] and for B3LYP/6-31G [and then the correction was added to the MP2/6-31G(d)//B3LYP/6-31G(d) values].

The effect of polar solvents was estimated by optimization of the equilibrium geometries and total energies with the implicit-solvent models included in the programs. The CPCM and SMD methods implemented in Gaussian 16 were tested (see Extended Scheme 2). As indicated in the corresponding Schemes, the comparisons were frequently carried out by means of Spartan'18 (CPCM); in some cases, SS(V)PE, a symmetrized version of IEF-PCM, and SM8 were used as well. Orbital drawings were also obtained from Spartan'18.

The stationary points were characterized by evaluating the harmonic vibrational frequencies at the optimized geometries to verify that minima and transition states have zero and one imaginary frequency, respectively.

We also carried out a few single-point calculations at higher levels, mainly at CCSD(T)/6-311+G(d,p), in order to check whether the energy differences (among the conformers or between reactions) predicted at the DFT level were maintained. Generally, this was the case.

It deserves comment that M06-2X predicts that reactions in Scheme 2 of the main text are more exothermic than CCSD(T)/6-311+G(d,p) does; however, the gaps are relatively small

[–29 kcal/mol vs. –28 kcal/mol with the CCSD(T)/6-311+G(d,p) for the addition of MeSH to *N*-methylmaleimide]

[–43 kcal/mol vs. –40.5 kcal/mol with the CCSD(T)/6-311+G(d,p) for the addition of MeSH to *N*-methylpropynamide],

so that for discussions and comparisons we always used the M06-2X results.

What matters are the relative reaction energies.

Complementary results

In Figure 1 of the main text, to locate each lowest-energy conformer, several species required a complete conformational analysis at an appropriate level of theory. A paradigmatic example is shown below (HC≡C–SO_2NHMe and the *Z* isomer of MeS–CH=CH–SO_2NHMe). A more complex case follows, where the presence of tetrahedrally substituted P atom, viz. the non-planar chiral structures of phosphonamidates HC≡C–PO(OMe)NHMe and MeS–CH=CH–PO(OMe)NHMe, increases the number of possible conformers. Only the most stable conformers are depicted in both cases. The relative energies, with regard to each lowest-energy conformer, are indicated in bold (in kcal/mol). As explained in the main text, the Δ*E* values were always obtained from the total energies of the corresponding lowest-energy conformers, for the sake of simplification.

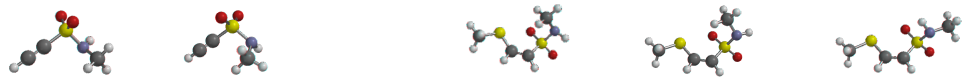

M06-2X/6-31G(d)	–720.37638 **1.1**	–720.37820 **0.0**	–1159.08838 **0.5**	–1159.08914 **0.0**	–1159.08672 **1.5**
M06-2X/6-311+G(d,p)[a]	–720.50646 **1.6**	–720.50901 **0.0**	–1159.26142 **0.0**	–1159.26121 **0.1**	–1159.25854 **1.8**
M06-2X/6-311+G(d,p)	–720.50671 **1.6**	–720.50921 **0.0**	–1159.26156 **0.0**	–1159.26136 **0.1**	–1159.25869 **1.8**
M06-2X/6-311+G(d,p)·w[b]	–720.52652 **2.2**	–720.53000 **0.0**	–1159.28746 **0.0**	–1159.28533 **1.3**	–1159.28295 **2.8**

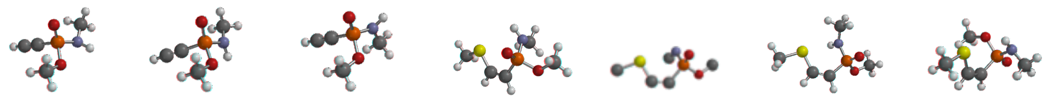

M06-2X/6-31G(d)	–703.56607 **1.3**	–703.56718 **0.6**	–703.56809 **0.0**	–1142.26656 **0.0**	–1142.26538 **0.7**	–1142.26482 **1.1**	–1142.26370 **1.8**
M06-2X/6-311+G(d,p)[a]	–703.71024 **1.7**	–703.71200 **0.6**	–703.71301 **0.0**	–1142.45191 **0.0**	–1142.45111 **0.5**	–1142.45044 **0.9**	–1142.45032 **1.0**
M06-2X/6-311+G(d,p)	–703.71055 **1.7**	–703.71234 **0.6**	–703.71324 **0.0**	–1142.45206 **0.0**	–1142.45136 **0.4**	–1142.45069 **0.9**	–1142.45051 **1.0**
M06-2X/6-311+G(d,p)·w[b]	–703.73110 **1.5**	–703.73318 **0.2**	–703.73355 **0.0**	–1142.47543 **0.1**	–1142.47555 **0.0**	–1142.47299 **1.5**	–1142.47415 **0.8**

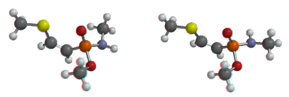

–1142.26440 **1.3**	–1142.26361 **1.8**
–1142.45005 **1.2**	–1142.44918 **1.7**
–1142.45026 **1.1**	–1142.44937 **1.6**
–1142.47293 **1.6**	–1142.47283 **1.5**

[a] Single-point calculations from M06-2X/6-31G(d)-optimized geometries.
[b] Optimized with water as the implicit solvent (CPCM, Spartan'18).

Figure S1. Total energies in au for the low-emergy conformers of (*N*-methyl)ethynesulfonamide, HC≡C–SO_2NHMe (upper row), and methyl (*N*-methyl)-phosphonamidate, HC≡C–PO(OMe)NHMe (lower row), and the corresponding adducts with MeSH. Relative energies in bold, in kcal/mol.

There are no significant differences between the M06-2X/6-311+G(d,p) and M06-2X/6-311+G(d,p)//M06-2X/6-31G(d) energies, as observed in similar substrates and commented above. It is worth noting that, for the adduct of MeSH and methyl (*N*-methyl)ethynephosphonamidate, there are several rotamers very close in energy.

Full Schemes of the main text

Extended Scheme 2. The energy values may be taken as an indirect evaluation of the performance of the different approaches regarding the addition reactions of thiols to activated double and triple bonds. It is observed, as partially commented above or in the main text, that: (1) there is a difference of 15 to 17 kcal/mol in passing from the total energy values to the ΔG° values; (2) M06-2X energies are more negative than CCSD(T)/6-311+G(d,p) energies (ca. –28 kcal/mol for the first reaction and –40.5 kcal/mol for the second reaction, which may be taken as the more reliable values), but the differences are relatively small; (3) at the SCS-MP2/6-311+G(d,p) level the predicted values (–27 and –37 kcal/mol) are less negative than those from the CCSD(T)/6-311+G(d,p) method; (4) when, from the frequency calculations, no corrections are introduced or an approximate scaling factor of 0.95 is included, the ΔH° and ΔG° values undergo changes of only 0.1–0.3 kcal/mol, so that no scaling corrections were considered in the remaining sections of the present work; (5) with different implicit-solvent models, the effect of a very polar solvent such as water was predicted to be insignificant (± 2 kcal/mol) for these reactions, which only involve neutral molecules (however, the SMD method affords ΔE values that are more different from the others); (6) the ωB97X method, with inclusion of dispersion corrections, afforded ΔE values very close to those of CCSD(T)/6-311+G(d,p) for the first reaction and to those of M06-2X for the second reaction; (7) M06-2X/6-311+G(d,p) and M06-2X/6-311+G(d,p)//M06-2X/6-31G(d) give almost the same total energies and reaction energies (differences of 0.0–0.1 kcal/mol); (8) the CCSD(T) total energies arising from B3LYP/6-31G(d) geometries are systematically lower than those arising from M06-2X geometries.

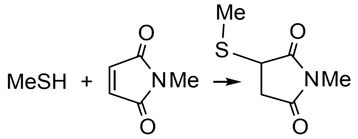

E MP2/6-31G(d)[a]	–437.95246 au	–397.57477 au	–835.57860 au	ΔE = –32.3 kcal/mol
G° MP2/6-31G(d)[a]	–437.93122	–397.51086	–835.47002	ΔG° = –17.5
E MP2/6-311+G(d,p)[a]	–438.03637	–397.78554	–835.87014	ΔE = –30.3
E SCS-MP2/6-31G(d)[a,b]	–437.94832	–397.55942	–835.55907	ΔE = –32.2
E SCS-MP2/6-311+G(d,p)[a,b]	–438.03963	–397.74771	–835.83035	ΔE = –27.0
E M06-2X[c]	–438.67517	–398.69859	–837.42007	ΔE = **–29.1**
G° M06-2X (without scaling)	–438.65373	–398.63231	–837.30759	ΔG° = –13.5
H° M06-2X (without scaling)	–438.62421	–398.59428	–837.25805	ΔH° = –24.8
G° M06-2X (scaling factor = 0.95)	–438.65609	–398.63735	–837.31552	ΔG° = –13.8
H° M06-2X (scaling factor = 0.95)	–438.62646	–398.59889	–837.26520	ΔH° = –25.0
E M06-2X·water/CPCM[d]	–438.68057	–398.71191	–837.43674	ΔE = –27.8
E M06-2X·water/SS(V)PE[d]	–438.68049	–398.71175	–837.43369	ΔE = –27.7
E M06-2X·water/CPCM[e]	–438.67898	–398.70844	–837.43197	ΔE = –27.0
E M06-2X·water/SMD[e]	–438.67698	–398.70994	–837.43487	ΔE = –30.1
E ωB97X-D/6-311+G(d,p)	–438.70624	–398.72371	–837.47432	ΔE = –27.8
E M06-2X//M06-2X/6-31G(d)	–438.67516	–398.69850	–837.41982	ΔE = –29.0
E CCSD(T)/6-31G(d)//M06-2X	–437.99318	–397.65317	–835.69325	ΔE = –29.4
E CCSD(T)/6-31+G(d)//B3LYP	–437.99677	–397.68047	–835.72486	ΔE = –29.9
E CCSD(T)/6-31+G(d)//M06-2X	–437.99666	–397.67852	–835.72345	ΔE = –30.3
E CCSD(T)/6-311+G(d,p)//B3LYP	–438.08019	–397.87161	–835.99636	ΔE = **–28.0**
E CCSD(T)/6-311+G(d,p)//M06-2X	–438.08025	–397.87053	–835.99606	ΔE = –28.4

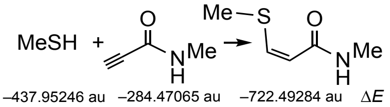

E MP2/6-31G(d)[a]	–437.95246 au	–284.47065 au	–722.49284 au	ΔE = –43.8 kcal/mol
G° MP2/6-31G(d)[a]	–437.93122	–284.41977	–722.39414	ΔG° = –27.0
E MP2/6-311+G(d,p)[a]	–438.03637	–284.63344	–722.73316	ΔE = –39.8
E SCS-MP2/6-31G(d)[a,b]	–437.94832	–284.45971	–722.47787	ΔE = –43.8
E SCS-MP2/6-311+G(d,p)[a,b]	–438.03963	–284.61083	–722.70966	ΔE = –37.1
E M06-2X[c]	–438.67517	–285.30679	–724.05057	ΔE = **–43.1**
G° M06-2X (without scaling)	–438.65373	–285.25194	–723.94828	ΔG° = –26.7
H° M06-2X (without scaling)	–438.62421	–285.21548	–723.90272	ΔH° = –39.6
G° M06-2X (scaling factor = 0.95)	–438.65609	–285.25632	–723.95550	ΔG° = –27.0
H° M06-2X (scaling factor = 0.95)	–438.62646	–285.21950	–723.90928	ΔH° = –39.7
E M06-2X·water/CPCM[d]	–438.68057	–285.32239	–724.07046	ΔE = –42.4
E M06-2X·water/SS(V)PE[d]	–438.68049	–285.32205	–724.06972	ΔE = –42.2
E M06-2X·water/CPCM[e]	–438.67898	–285.31825	–724.06529	ΔE = –42.7
E M06-2X·water/SMD[e]	–438.67698	–285.31981	–724.06843	ΔE = –45.0
E ωB97X-D/6-311+G(d,p)	–438.70624	–285.32683	–724.10154	ΔE = –43.0
E M06-2X//M06-2X/6-31G(d	–438.67516	–285.30664	–724.05047	ΔE = –43.1
E CCSD(T)/6-31G(d)//B3LYP	–437.99325	–284.53550	–722.59902	ΔE = –44.1
E CCSD(T)/6-31G(d)//M06-2X	–437.99318	–284.53433	–722.59811	ΔE = –44.3
E CCSD(T)/6-311+G(d,p)//B3LYP	–438.08019	–284.70292	–722.84754	ΔE = **–40.4**
E CCSD(T)/6-311+G(d,p)//M06-2X	–438.08025	–284.70212	–722.84702	ΔE = –40.6

[a] Single-point calculations from B3LYP/6-31G(d)-optimized geometries.
[b] Calculations with ORCA.
[c] M06-2X = M06-2X/6-311+G(d,p), throughout this work.
[d] Optimization in water with Spartan'18.2. With the SM8 model (with a minimal basis set), ΔE = –31.7 kcal/mol for the first addition reaction.
[e] Optimization in water with Gaussian 16.

Extended Scheme 3. The total energies that appear in the first row are directly obtained by the M06-2X/6-311+G(d,p) method, while those in the second row have been optimized in water as the implicit solvent (CPCM) with the same method. They belong to the lowest-energy conformer of each molecule. This was determined after a standard conformational search by empirical methods, selection of the low energy species from M06-2X/6-31G(d) or MP2/6-31G(d)//B3LYP/6-31(G), and final ordering by means of M06-2X/6-311+G(d,p) or M06-2X/6-311+G(d,p)·w (CPCM), as just mentioned. Not always the lowest-energy conformer of the isolated species (gas phase, in vacuo) was coincident with that obtained in water; such cases are commented in due course.

The reaction energies are given in kcal/mol. All the reactions are exoergic (around –24 kcal/mol for the maleimide, around –40 for the activated triple bonds, in accordance with what was observed for the addition of MeSH). We assume that the corresponding enthalpy energies (Gibbs free energies) would generally be ca. 16 kcal/mol less favorable.

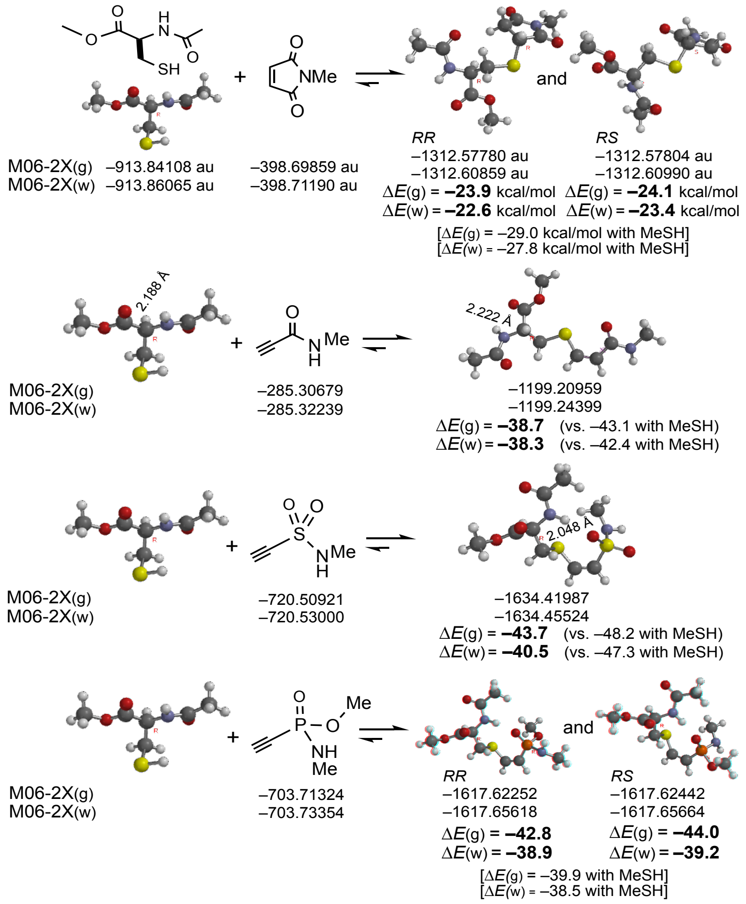

The phosphonamidate case is also included in the expanded Scheme 3. The energy difference between the *RR* and *RS* stereoisomers is predicted to be small, in water. As indicated in Figure S1, there are several conformers very close in energy (a much higher number than in any other case examined by us), due to the tetrahedral arrangement of the substituents bound to the P atom. Another feature is that the energy difference between the adduct of MeSH and that of AcCysOMe was in this case reversed, which we attribute to the network of intramolecular hydrogen bonds (involving the P=O, NHAc, CO groups, NHMe, and OMe groups) that occur in the latter and overstabilize it, especially in the gas phase. Actually, the phosphonamidate case (alkynephosphonamidates, alkenephosphonamidates, allenephosphonamidates...) would require an independent study.

Extended Figure 2. The M06-2X-predicted values for the reaction of addition of MeS^- (methanethiolate ion) with optimization in water were collected in Figure 2 of the main text, the details of which are included below. This Figure can be expanded up to 40–50 pairs as we did for the equilibria involving neutral species (Figure 1), but the selection of samples is illustrative of the changes that occur when the anion (MeS^-) is involved instead of MeSH: the activated double bonds follow a pattern different from that of activated triple bonds. As pointed out in the main text, the energy for the reaction with MeSH is indicative of the thermodynamic stability of the adduct with regard to the retro-Michael reaction (the corresponding E2 mechanism can be favored under basic catalysis and on heating), whereas the energy for the reaction with the anion to give an anionic intermediate is indicative of the kinetics of the process (the lower the energy of such an intermediate, the lower the barrier to reach it).

The total energies in au or Hartrees as directly obtained by M06-2X, that is, by optimization of geometries at the M06-2X/6-311+G(d,p) level, are given in the following extended Figure 2 for each Michael acceptor in the gas phase and optimized in w (CPCM/Spartan'18, in blue), which are the two upper values, and for each adduct, which are the two lower values.

ΔE in kcal/mol, from M06-2X/6-311+G(d,p), in water (in blue) and in the gas phase, for the addition reaction of CH_3S^- to the depicted acceptors

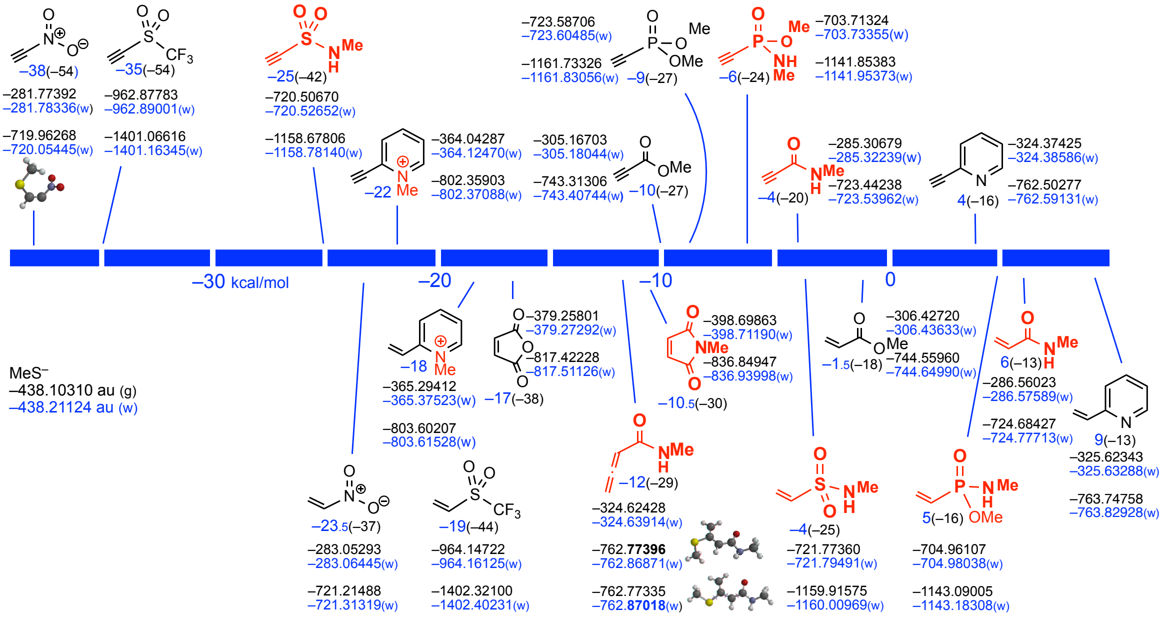

Quite often the lowest-energy anionic adducts adopted a conformation with the MeS folded (due to the electrostatic interaction between the Me group and the partially delocalized negative charge), whereas in water a zigzag conformation (*ap*) of the SMe group turned out often to be that of lowest energy.

Since, as indicated in the main text, the reliability of some implicit-solvent models is a hot topic under debate, especially when charged molecules are involved, we include below the scale in the gas phase. The relative values are essentially maintained, with a few exchanges of positions (in the next Figure, as shown by the crossing lines, with regard to the preceding Figure, which is Figure 2 of the main text). This may be understood assuming that disparate delocalized anions will not be similarly stabilized by a polar solvent.

ΔE in kcal/mol, from M06-2X/6-311+G(d,p) for the addition reaction of CH_3S^- to the depicted acceptors in the gas phase

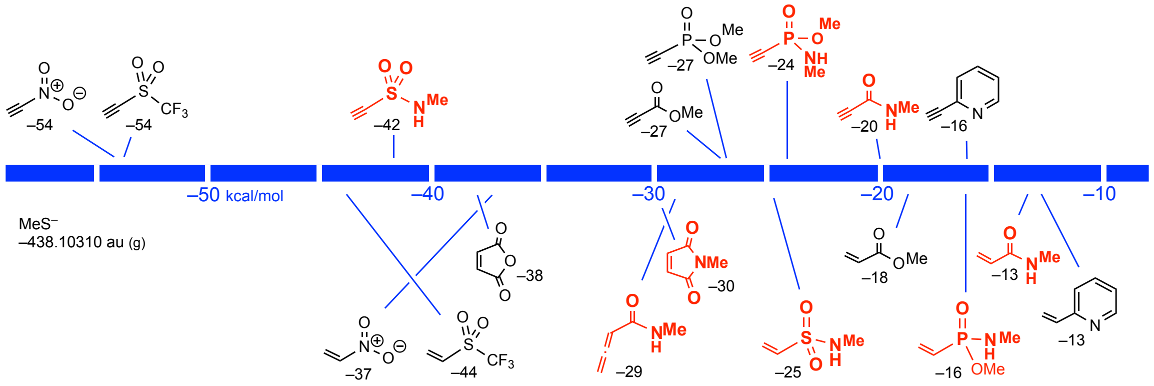

Finally, the following Figure shows the reaction energies in water/CPCM, obtained with Gaussian 16. Despite the fact that the total energies were different, there are almost no changes of position with regard to the first Figure, which indicates that the CPCM parameters implemented in the two packages are qualitatively consistent.

ΔE in kcal/mol, from M06-2X/6-311+G(d,p) in water (CPCM/Gaussian) for the addition reaction of CH_3S^- to the depicted acceptors

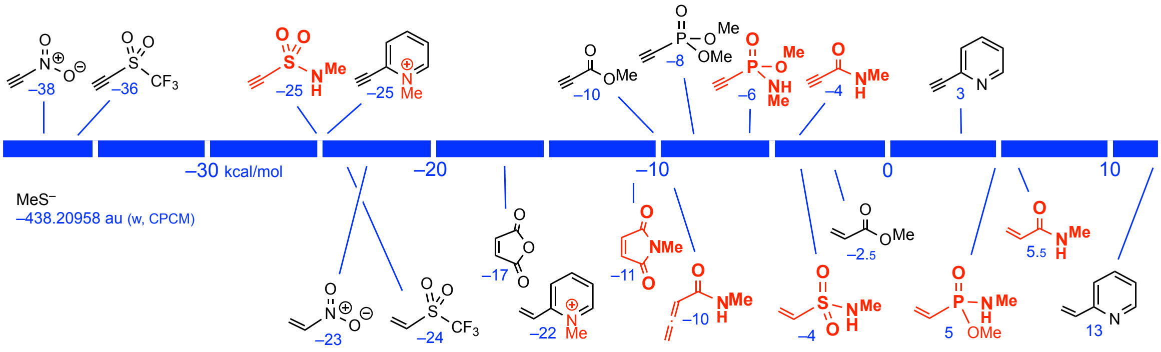

Extended Scheme 9. Kinetically, the second addition of MeS⁻ to *N*-methylpropynamide is unfavorable (see Δ*E*(w) = 6.6 kcal/mol in Scheme 9, hence Δ*G*°(w) 22–23 kcal/mol, and see Δ*G*‡(w) = 25 kcal/mol; it is even more unfavorable for the case of ^{t}BuS⁻ (see Scheme 6). Simple steric and electronic effects explain these differences.

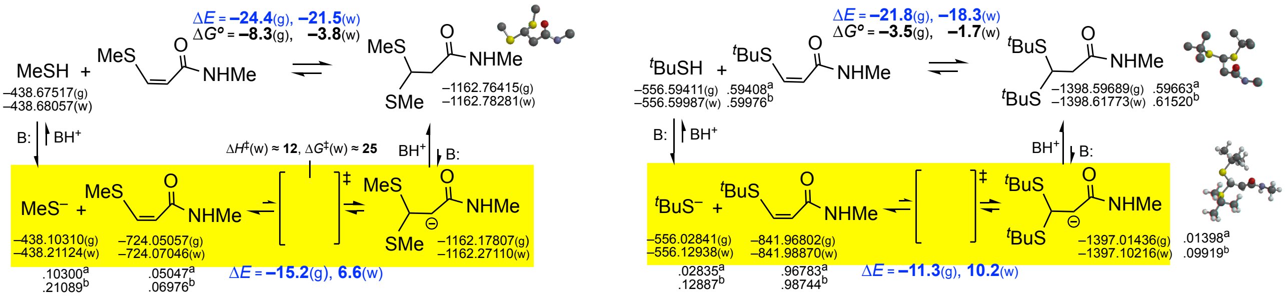

M06-2X total energies are in au. Values at the M06-2X/6-311+G(d,p)//M06-2X/6-31G(d) level[a] and single-point calculations in water (CPCM, Spartan'18) M06-2X/6-311+G(d,p)·w//M06-2X/6-31G(d)[b] are also indicated in several cases, for the sake of comparison: it is observed that the differences are small. Reaction energies are in kcal/mol, as always.

Extended Scheme 10. The M06-2X total energies for the lowest-energy species involved in the first addition of MeSH to representative triple bonds and in the second addition are given in au. The reaction energies obtained from these values are in kcal/mol. In all cases, the second addition, as expected, is predicted to be many kcal/mol less feasible.

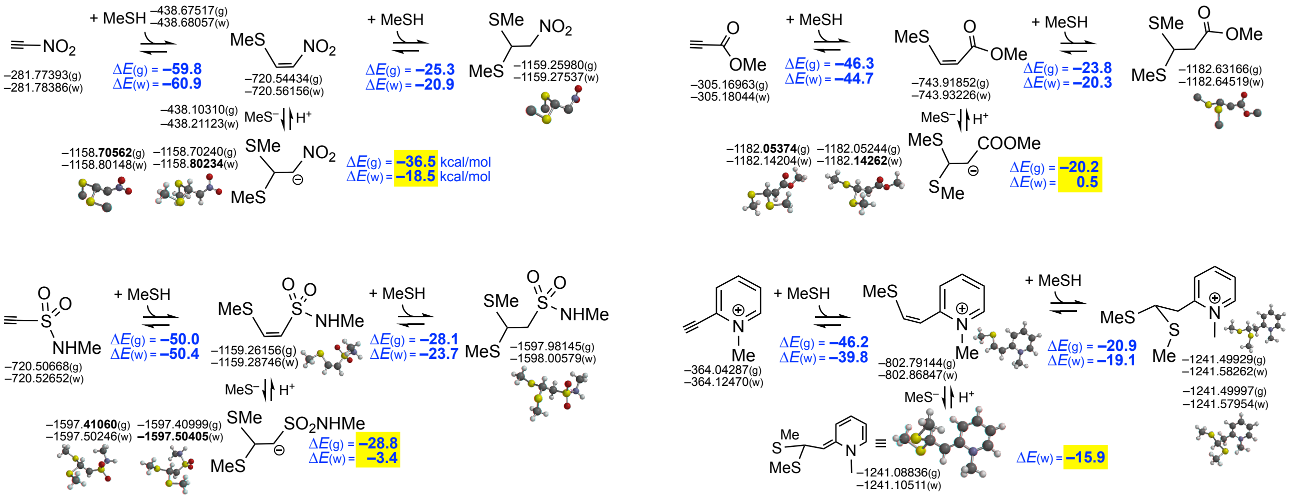

Relevant M06-2X/6-311+G(d,p) equilibrium geometries (Cartesian coordinates)

The Cartesian coordinates (in Å) of the lowest energy conformers for a selection of the more relevant molecular entities and anionic species are given below.

N-methylmaleimide

1	C	C3	-0.3276215	-0.0034945	1.1438436
2	C	C1	-1.7546204	-0.0220985	0.6641020
3	H	H2	-2.5854101	-0.0323085	1.3542414
4	C	C2	-1.7546202	-0.0220841	-0.6641021
5	H	H3	2.5854098	-0.0322791	-1.3542418
6	C	C4	-0.3276213	-0.0034697	-1.1438432
7	O	O1	0.0743214	0.0016493	2.2750744
8	N	N1	0.4622843	0.0126216	0.0000004
9	O	O2	0.0743221	0.0016987	-2.2750739
10	C	C5	1.9111777	0.0192575	-0.0000004
11	H	H1	2.3076646	-0.9974527	-0.0000195
12	H	H4	2.2527663	0.5389634	0.8938829
13	H	H5	2.2527669	0.5389969	-0.8938637

1-methyl-3-(methylsulfanyl)succinimide

1	C	C3	0.3570420	0.2911346	1.8834561
2	C	C4	0.4104029	-0.5406036	-0.2806177
3	O	O1	0.7847772	0.7119151	2.9234604
4	N	N1	1.1436663	-0.1036316	0.8092239
5	O	O2	0.8836870	-0.9957618	-1.2896553
6	C	C1	-1.0643819	-0.3105169	0.0207740
7	H	H3	-1.6367300	-1.2052993	-0.2241494
8	C	C2	-1.0963883	0.0641872	1.5044720
9	H	H2	-1.4845763	-0.7467781	2.1235920
10	H	H9	-1.6759677	0.9603797	1.7278116
11	C	C5	2.5939377	-0.0707578	0.8332592
12	H	H1	2.9934757	-1.0045292	1.2316715
13	H	H4	2.9059764	0.7550967	1.4702933
14	H	H5	2.9510050	0.0689627	-0.1854490
15	S	S1	-1.6839603	1.0833106	-0.9819559
16	C	C6	-1.5971791	0.3563767	-2.6409138
17	H	H6	-0.5691155	0.1339339	-2.9168362
18	H	H7	-2.0123065	1.1010333	-3.3193815
19	H	H8	-2.2033646	-0.5484522	-2.6890549

TS, MeS$^-$ + *N*-methylmaleimide in water (CPCM)

1	C	-1.74746	-0.72616	-0.05523
2	C	-0.08231	0.65844	0.69458
3	O	-2.79246	-1.00920	-0.63073
4	N	-1.21871	0.58430	-0.07999
5	O	0.49191	1.68152	1.02064
6	C	-1.88340	1.70821	-0.69974
7	H	-2.86834	1.86897	-0.25775
8	H	-1.99932	1.53748	-1.77075
9	H	-1.26760	2.59143	-0.53893
10	C	-0.85869	-1.53088	0.74835
11	C	0.22213	-0.75518	1.10151
12	H	-1.00850	-2.58479	0.91919
13	H	0.93808	-0.94536	1.88548
14	S	1.91065	-0.84661	-0.63035
15	C	3.10558	0.51279	-0.39761
16	H	3.51834	0.81706	-1.36085
17	H	3.92609	0.21763	0.25715
18	H	2.59100	1.37131	0.04158

HC≡C–CONHMe, *N*-methylpropynamide

1	C	C3	-0.4222587	-0.6470120	0.0000000
2	C	C1	-1.8165404	-0.1866120	0.0000000
3	C	C2	-2.9634089	0.1633030	0.0000000
4	H	H3	-3.9854308	0.4623826	0.0000000
5	O	O1	-0.1348176	-1.8236272	0.0000000
6	C	C4	1.9097601	0.1407156	0.0000000
7	H	H4	2.0717182	-0.9351949	0.0000000
8	H	H5	2.3712381	0.5706627	0.8906967
9	H	H6	2.3712381	0.5706627	-0.8906967
10	N	N1	0.4775506	0.3711660	0.0000000
11	H	H1	0.1209514	1.3135533	0.0000000

MeS–CH=CH–CONHMe, *Z*

1	C	C3	1.0423954	0.0660454	0.0000010
2	O	O1	0.7986499	1.2625309	0.0000016
3	C	C4	3.4387693	0.5189250	-0.0000002
4	H	H4	4.3667648	-0.0502899	-0.0000012
5	H	H5	3.4067903	1.1597667	0.8831877
6	H	H6	3.4067887	1.1597670	-0.8831878
7	C	C1	-0.0202567	-0.9595455	0.0000006
8	C	C2	-1.3209416	-0.6362492	-0.0000011
9	H	H2	0.2555093	-2.0084934	0.0000020
10	H	H1	-2.0563950	-1.4357203	-0.0000008
11	S	S1	-2.0048668	0.9659983	-0.0000033
12	C	C5	-3.7603672	0.4905369	-0.0000004
13	H	H3	-4.0157696	-0.0765039	-0.8948547
14	H	H7	-4.3301126	1.4192805	0.0000020
15	H	H8	-4.0157656	-0.0765059	0.8948538
16	N	N1	2.3214241	-0.4040105	0.0000006
17	H	H9	2.4873836	-1.3955320	0.0000003

TS, MeS$^-$ + HC≡C–CONHMe in water (CPCM)

1	C	1.59786	0.54929	0.06474
2	O	2.08451	1.01345	1.10277
3	C	3.44312	-1.05184	-0.13521
4	H	3.77198	-1.79253	-0.86099
5	H	3.30386	-1.53950	0.83229
6	H	4.21748	-0.29008	-0.02832
7	C	0.37998	1.03795	-0.52229
8	C	-0.85133	0.99137	-0.64196
9	H	-1.74091	1.39324	-1.09179
10	S	-2.22995	-0.63059	0.52401
11	C	-3.79796	-0.13848	-0.26886
12	H	-3.63445	0.71223	-0.94151
13	H	-4.53849	0.16676	0.47107
14	H	-4.21648	-0.95121	-0.86342
15	N	2.20702	-0.46009	-0.60952
16	H	1.76096	-0.82611	-1.43542

MeS-CH=C$^-$–CONHMe, *Z*, anion, MeS *folded*

1	C	C3	0.1649251	-0.8585924	0.3076391
2	O	O1	0.4867226	-1.0017754	1.4975935
3	C	C4	2.5280519	-0.4868304	-0.2365482
4	H	H4	2.8621011	-1.5325671	-0.3106997
5	H	H5	2.6696563	-0.1702483	0.7958946
6	H	H6	3.1524851	0.1307404	-0.8876103
7	C	C1	-1.0300894	-1.2709589	-0.3247513
8	C	C2	-2.1728496	-0.7121455	-0.6469574
9	N	N1	1.1328248	-0.3054326	-0.5840339
10	H	H9	0.9105991	-0.5236971	-1.5450524
11	S	S1	-2.7872528	0.9947285	-0.3122919
12	C	C5	-1.4339922	1.6399531	0.7028036
13	H	H3	-1.2772577	0.9948880	1.5672865
14	H	H7	-1.7205988	2.6419993	1.0256844
15	H	H8	-0.5085894	1.6829800	0.1283949
16	H	H11	-2.9767360	-1.2230414	-1.1773517

MeS-CH=C$^-$–CONHMe, *Z*, anion, in w, MeS *ap*

1	C	C3	0.9928244	0.6613592	0.7528759
2	O	O1	1.1858447	1.8811871	0.5646995
3	C	C4	3.1002448	0.1588523	-0.4218603
4	H	H4	3.6186890	-0.7318004	-0.7730931
5	H	H5	3.7482266	0.6956722	0.2775152
6	H	H6	2.9065619	0.8097787	-1.2756371
7	C	C1	-0.0749348	0.1296161	1.5811566
8	C	C2	-1.1946857	-0.3099679	1.0163078
9	H	H1	-2.0443737	-0.7192875	1.5651420
10	S	S1	-1.5322302	-0.3931423	-0.7775195
11	C	C5	-3.3381279	-0.2041927	-0.7546270
12	H	H3	-3.7789642	-0.9384663	-0.0803913
13	H	H7	-3.7045267	-0.3862432	-1.7641425
14	H	H8	-3.6208408	0.8003483	-0.4425874
15	N	N1	1.8566280	-0.2469888	0.2056081
16	H	H9	1.7477787	-1.2108802	0.4798705

MeS-CH=C$^-$–CONHMe, *E*, anion, MeS *folded*

1	C	C3	0.6893248	0.2185319	0.9192997
2	O	O1	0.9234180	0.1670449	2.1461261
3	C	C4	2.9120510	1.2268157	0.5696569
4	H	H4	3.4829365	0.3627646	0.9228316
5	H	H5	2.8357653	1.9425104	1.3885786
6	H	H6	3.4528162	1.6950034	-0.2514040
7	C	C1	-0.4547969	-0.3985029	0.2770581
8	C	C2	-1.4697352	0.3974901	-0.0453975
9	N	N1	1.5914456	0.8489159	0.0984257
10	H	H9	1.4581143	0.7387363	-0.8947198
11	S	S2	-3.0073632	-0.1046672	-0.8453758
12	C	C6	-2.7524465	-1.8787859	-1.0676886
13	H	H2	-1.8738236	-2.0604239	-1.6865665
14	H	H1	-3.6428205	-2.2668729	-1.5609566
15	H	H10	-2.6181498	-2.3660682	-0.1026214
16	H	H12	-1.5267358	1.4775077	0.1327536

(MeS)$_2$CHCH$_2$CONHMe

1	C	C4	0.3197957	-0.2907827	1.0674886
2	C	C5	1.6306526	-1.1649452	-1.2507514
3	C	C1	-1.3946214	1.0123313	-0.1924491
4	O	O1	-1.3911893	2.0017116	0.5128215
5	S	S1	0.5756617	-1.8651565	1.9521776
6	C	C6	2.0582162	-1.4418653	2.9001464
7	H	H13	0.2815103	0.5208111	1.7964081
8	S	S2	1.6808256	0.1933933	-0.0519584
9	C	C3	-1.0310552	-0.3576793	0.3582781
10	H	H1	-1.7996113	-0.6290626	1.0878771
11	H	H4	-1.0233928	-1.1275961	-0.4174328
12	H	H26	1.8549249	-0.6123861	3.5779819
13	H	H27	2.8762034	-1.1855807	2.2280551
14	H	H28	2.3236154	-2.3234265	3.4822055
15	H	H29	0.7950626	-1.0613969	-1.9435460
16	H	H30	1.5774929	-2.1220582	-0.7302191
17	H	H31	2.5602053	-1.1168146	-1.8163276
18	C	C2	-2.0585061	2.3284525	-2.1291011
19	H	H2	-2.9395356	2.7702760	-1.6606093
20	H	H5	-2.2567864	2.1620717	-3.1864160
21	H	H6	-1.2267994	3.0263308	-2.0178039
22	N	N1	-1.7332061	1.0591008	-1.5045109
23	H	H3	-1.6794634	0.2242720	-2.0623144

HC≡C–SO$_2$NHMe, (*N*-methyl)ethynesulfonamide

1	C	C1	-1.4539424	0.0740155	-0.8807501
2	C	C2	-2.4715476	0.7023285	-0.9588661
3	H	H3	-3.3803410	1.2546652	-1.0237496
4	S	S1	0.0257093	-0.8277415	-0.8442163
5	O	O2	0.9348108	-0.1395042	-1.7272629
6	O	O1	-0.2801441	-2.2254162	-1.0127015
7	N	N1	0.4681327	-0.6274826	0.7315437
8	H	H2	0.8323113	-1.5014479	1.0940186
9	C	C3	1.2067369	0.5877355	1.0795668
10	H	H1	0.6052726	1.4536743	0.8026528
11	H	H4	2.1741320	0.6503335	0.5797698
12	H	H5	1.3388696	0.5988397	2.1599947

MeS–CH=CH–SO$_2$NHMe, *Z*

1	S	S1	0.8567862	1.2155825	-1.1331280
2	C	C5	1.5016016	0.8323267	-2.7885137
3	S	S2	-0.0286024	0.2003297	2.0242259
4	O	O2	-0.0308916	1.5739162	1.5659736
5	O	O1	0.4008246	-0.1822491	3.3513656
6	N	N1	-1.5891270	-0.3412675	1.8867233
7	C	C1	0.9236326	-0.7298536	0.8640607
8	H	H2	1.2455543	-1.6943971	1.2360089
9	C	C2	1.2344258	-0.3063594	-0.3654089
10	H	H16	1.8189153	-0.9800705	-0.9869302
11	H	H15	2.5673988	0.6102796	-2.7492302
12	H	H17	1.3534326	1.7312026	-3.3851419
13	H	H18	0.9542622	0.0102808	-3.2481412
14	C	C4	-2.2429058	-0.2111074	0.5857933
15	H	H1	-2.1713091	0.8277473	0.2651370
16	H	H3	-3.2944499	-0.4639320	0.7088374
17	H	H4	-1.7990775	-0.8558332	-0.1806035
18	H	H7	-1.7004707	-1.2365956	2.3489718

TS, MeS$^-$ + HC≡C–SO$_2$NHMe in water (CPCM)

1	C	-0.04268	0.86462	-0.16568
2	C	0.94397	1.58884	-0.23735
3	S	3.26488	0.52860	-0.02750
4	C	2.58427	-1.11779	0.34413
5	H	3.08665	-1.56935	1.20079
6	H	1.52082	-1.02768	0.58891
7	H	2.67680	-1.79452	-0.50680
8	H	1.43649	2.53013	-0.34752
9	S	-1.07906	-0.47093	-0.08283
10	O	-0.75192	-1.41598	-1.14092
11	O	-1.12360	-0.93015	1.29848
12	N	-2.60615	0.06408	-0.43491
13	H	-2.67009	0.27165	-1.42808
14	C	-3.13783	1.13347	0.41727
15	H	-4.14825	1.35296	0.07814
16	H	-2.53195	2.04349	0.36902
17	H	-3.18271	0.77636	1.44462

MeS–CH=C$^-$–SO$_2$NHMe, *Z*, anion, MeS *folded*

1	C	C	1.1531136	-0.2604007	1.1716319
2	C	C	2.3464456	0.0028866	0.6393113
3	S	S	-0.2990438	-0.0406480	0.2599388
4	O	O	-0.4814764	1.1995127	-0.5158614
5	O	O	-0.6962381	-1.2763383	-0.4503581
6	N	N	-1.3768570	0.1596579	1.5678749
7	H	H	-1.1238742	-0.5519512	2.2465853
8	C	C	-2.7743939	0.0930542	1.1548804
9	H	H	-3.4057686	0.3029985	2.0207590
10	H	H	-3.0523977	-0.8740882	0.7198275

11	H	H	-2.9433434	0.8709791	0.4082343
12	H	H5	3.2211874	-0.1215921	1.2790727
13	S	S1	3.0086616	0.5424042	-0.9533866
14	C	C1	1.7775209	0.0667450	-2.1983127
15	H	H2	1.3429442	-0.8978423	-1.9336017
16	H	H3	2.3195680	-0.0207919	-3.1413802
17	H	H4	0.9839519	0.8054143	-2.2752155

MeS–CH=C$^-$–SO_2NHMe, *Z*, anion, w, MeS *ap*

1	C	C	-0.4462935	-1.0630648	0.2128240
2	C	C	-1.6029947	-0.4342785	-0.0323903
3	S	S	0.9612043	-0.0469312	0.1505235
4	O	O	1.0748325	0.8963667	-0.9861284
5	O	O	1.2060020	0.5948983	1.4658526
6	N	N	2.1611809	-1.1858320	-0.1446300
7	H	H	2.0124772	-1.9650241	0.4923310
8	C	C	3.5278643	-0.6544009	-0.0631164
9	H	H	4.2155596	-1.4644354	-0.3014376
10	H	H	3.7695706	-0.2556944	0.9263284
11	H	H	3.6477337	0.1321696	-0.8080949
12	H	H5	-2.5339807	-0.9977550	0.0293452
13	S	S1	-1.9565200	1.2701043	-0.4867345
14	C	C1	-3.7427363	1.2798726	-0.1558210
15	H	H2	-4.2366662	0.4992352	-0.7340468
16	H	H3	-4.1185520	2.2522679	-0.4714159
17	H	H4	3.9386818	1.1425017	0.9066111

$(MeS)_2CHCH_2SO_2$NHMe

1	S	S1	-0.8902532	1.6523977	-1.8049769
2	C	C5	-2.3716523	1.1978120	-2.7393295
3	S	S2	0.6259956	-0.9347496	1.3740503
4	O	O2	1.6606526	-1.8149300	0.8648453
5	O	O1	-0.6829414	-1.4350727	1.7511355
6	N	N1	1.3252046	-0.1313485	2.6611661
7	H	H15	-2.1859357	0.2868261	-3.3067494
8	H	H17	-2.5780664	2.0195065	-3.4237875
9	H	H18	-3.2221352	1.0632796	-2.0708844
10	C	C4	0.4270970	0.3528802	3.7128032
11	H	H1	-0.2720041	1.0725657	3.2848956
12	H	H3	1.0325813	0.8720360	4.4547500
13	H	H4	-0.1433777	-0.4483705	4.1860313
14	H	H7	2.1060034	-0.6889894	2.9923186
15	C	C1	0.3646920	0.4331387	0.2322269
16	H	H2	0.0487917	1.2801659	0.8450873
17	H	H6	1.3405110	0.6566948	-0.2018712
18	C	C2	-0.6975962	0.1231168	-0.8153070
19	H	H10	-1.6407812	-0.0775411	-0.3030175
20	S	S3	-0.3897006	-1.3856405	-1.7846774
21	C	C3	1.2913535	-1.0575794	-2.3753419
22	H	H5	1.3532107	-0.0486467	-2.7864780
23	H	H8	1.4831492	-1.7753332	-3.1715423
24	H	H9	2.0152014	-1.2122186	-1.5753472

HC≡C–PO(OMe)NHMe, methyl (*N*-methyl)ethyne phosphonamidate

1	C	C	-0.3967409	-1.5410127	0.2954344
2	C	C	-1.2319640	-2.3551053	0.5895250
3	H	H	-1.9630264	-3.0829302	0.8558860
4	P	P	0.8499236	-0.3658837	-0.1790877
5	O	O	2.0149331	-0.9677766	-0.8461541
6	O	O	-0.0093339	0.6689189	-1.0610700
7	C	C	-0.5371768	0.2319439	-2.3171352
8	H	H	0.2638796	-0.1581010	-2.9470427
9	H	H	-0.9880933	1.1049239	-2.7842091
10	H	H	-1.2982099	-0.5360551	-2.1602085
11	N	N	1.1818561	0.4801221	1.1888478
12	H	H	2.1500072	0.7524371	1.2794399
13	C	C	0.1879688	1.2961342	1.8799217
14	H	H	-0.7510918	0.7422314	1.9348460
15	H	H	0.0030922	2.2462099	1.3720451
16	H	H	0.5239765	1.4839431	2.8989612

MeS–CH=CH–PO(OMe)NHMe, *Z*

1	C	C1	-0.3196264	-1.1892951	-0.6991874
2	C	C2	0.0883607	-1.8654217	0.3785529
3	H	H2	-1.0964407	-1.6136984	-1.3252295
4	H	H1	-0.3656212	-2.8231024	0.6200633
5	S	S1	1.3653845	-1.3795829	1.4783343
6	C	C5	1.1218553	-2.6211712	2.7794993
7	H	H3	0.1498509	-2.5043756	3.2557727
8	H	H7	1.9042878	-2.4531727	3.5177173
9	H	H8	1.2317943	-3.6251083	2.3713713
10	P	P1	0.3667270	0.3859157	-1.2224221
11	O	O1	1.7010081	0.3751165	-1.8573343
12	O	O2	-0.8576739	0.8611605	-2.1587337
13	C	C3	-0.6848069	2.0691897	-2.9050361
14	H	H4	0.1772465	1.9819014	-3.5681116
15	H	H6	-0.5427290	2.9145472	-2.2274580
16	H	H9	-1.5933055	2.2081625	-3.4864777
17	N	N1	0.3237032	1.4481429	0.0600009
18	C	C6	-0.8209440	1.5285459	0.9596703
19	H	H5	-0.7799878	2.4649381	1.5166672
20	H	H13	-0.8601962	0.6923637	1.6665770
21	H	H14	-1.7371000	1.5291515	0.3668811
22	H	H15	1.2282134	1.6157928	0.4788830

TS, MeS$^-$ + HC≡C–PO(OMe)NHMe in water (CPCM)

1	C	-0.00407	-1.01704	1.18727
2	C	-1.24378	-1.06064	1.14774
3	H	-2.17631	-1.48135	1.47564
4	S	-2.57345	0.40567	-0.36073
5	C	-4.00432	-0.72769	-0.41296
6	P	1.27911	-0.11606	0.42615
7	O	2.28814	0.47799	1.34905
8	O	1.86075	-1.23186	-0.58089
9	C	2.99841	-0.90050	-1.38939
10	H	2.77486	-0.04297	-2.02562
11	H	3.20666	-1.77312	-2.00331
12	H	3.85978	-0.68009	-0.75704
13	N	0.65870	1.04545	-0.60469
14	H	-0.14142	0.68619	-1.12501
15	C	0.36781	2.37425	-0.04881
16	H	1.26458	2.76470	0.42977
17	H	-0.44842	2.35560	0.68108
18	H	0.09109	3.04257	-0.86373
19	H	-4.93959	-0.18082	-0.29024
20	H	-3.94440	-1.46283	0.39754
21	H	-4.04460	-1.27528	-1.35514

MeS–CH=C$^-$–PO(OMe)NHMe, *Z*, anion, MeS *folded*

1	C	C1	-1.2489505	-0.7736332	-0.9417176
2	C	C2	-1.0855983	-2.0693739	-0.7525997
3	H	H1	-1.6909189	-2.8332785	-1.2387841
4	S	S1	0.1725194	-2.9588962	0.3242823
5	C	C5	1.6945900	-2.4739814	-0.5529012
6	H	H3	1.7235405	-2.9422929	-1.5369871
7	H	H7	2.5458132	-2.8177084	0.0374254
8	H	H8	1.7389542	-1.3903687	-0.6676992
9	P	P1	-0.3076874	0.4848928	-0.1943942
10	O	O1	0.9370562	0.9194880	-0.9152234
11	O	O2	-1.3082752	1.7854001	-0.0560595
12	C	C3	-1.6217962	2.4469799	-1.2668510
13	H	H4	-0.7128249	2.8194566	-1.7487488
14	H	H6	-2.2783503	3.2828164	-1.0179643
15	H	H9	-2.1366466	1.7631085	-1.9490558
16	N	N1	-0.1118241	0.2131940	1.4624120
17	H	H5	0.0904901	-0.7664246	1.6367932
18	C	C4	0.7435172	1.1367378	2.1962420
19	H	H10	0.3694782	2.1517543	2.0486104
20	H	H11	1.7899245	1.1111135	1.8683766
21	H	H12	0.6969890	0.9110159	3.2648439

MeS–CH=C$^-$–PO(OMe)NHMe, *Z*, anion, w, MeS *ap*

1	C	C1	0.2105933	-0.0220584	-1.8227916
2	C	C2	0.0070144	-1.2805517	-1.4412245
3	H	H1	-0.3680872	-2.0431179	-2.1293757
4	S	S1	0.2557875	-2.0799518	0.1795426
5	C	C5	-0.3970970	-3.7270348	-0.2243350
6	H	H3	-1.4370948	-3.6597070	-0.5439237
7	H	H7	-0.3413701	-4.3281090	0.6824699
8	H	H8	0.2009406	-4.1988202	-1.0038020
9	P	P1	0.7250697	1.2786486	-0.8022461
10	O	O1	1.7247664	2.2304758	-1.4049672
11	N	N1	-0.6169310	2.1632695	-0.2817030
12	H	H2	-0.9317050	2.8179419	-0.9898058
13	C	C3	-1.7223430	1.4298969	0.3405007
14	H	H4	-1.3388153	0.8712303	1.1970692
15	H	H5	-2.2029230	0.7214343	-0.3435855
16	H	H6	-2.4687170	2.1374182	0.7010814
17	O	O2	1.2825933	0.6857465	0.6201630
18	C	C4	1.7616954	1.5874615	1.6210447
19	H	H9	2.6113081	2.1620436	1.2443999
20	H	H10	2.0780453	0.9836905	2.4700198
21	H	H11	0.9672697	2.2700932	1.9314688

$(MeS)_2CHCH_2$PO(OMe)NHMe

1	C	C4	0.7182787	1.0260171	-0.9066481
2	C	C5	2.5240324	2.7385414	-2.0975046
3	S	S1	2.0815822	-0.0192723	-0.2509718
4	C	C6	2.4413913	0.8741965	1.2928688
5	H	H13	0.5862006	1.8427590	-0.1938508
6	S	S2	1.0924920	1.7294533	-2.5446275
7	C	C3	-0.5906633	0.2476550	-1.0198776
8	H	H1	-0.4823870	-0.6041174	-1.6975174
9	H	H4	-1.3762737	0.8939814	-1.4201851
10	H	H26	1.5428110	0.9426929	1.9060225
11	H	H27	2.8333997	1.8674831	1.0723040
12	H	H28	3.2078642	0.3006157	1.8132878
13	H	H29	3.3179065	2.1036113	-1.7018721
14	H	H30	2.2441306	3.5018449	-1.3689180
15	H	H31	2.8708098	3.2256421	-3.0091277
16	P	P1	-1.1572646	-0.3208257	0.6126932
17	O	O1	-0.8312264	0.6167188	1.7140108
18	O	O2	-2.7169599	-0.5356794	0.2715331
19	C	C1	-3.5869390	-0.9677243	1.3242083
20	H	H2	-3.3339577	-1.9851230	1.6289888
21	H	H5	-4.5970710	-0.9375793	0.9230193
22	H	H6	-3.5043567	-0.2957566	2.1801133
23	N	N1	-0.6430784	-1.8692459	0.9608546
24	H	H3	0.2102646	-1.8648741	1.5040802
25	C	C2	-0.6916094	-2.9188769	-0.0547641
26	H	H7	-0.6357333	-3.8960124	0.4281068
27	H	H8	0.1222166	-2.8374846	-0.7846116
28	H	H9	-1.6458602	-2.8586407	-0.5816148

AcCysOMe + *N*-methylmaleimide, *RS*

1	C	C4	0.7182787	1.0260171	-0.9066481
2	C	C5	2.5240324	2.7385414	-2.0975046
3	S	S1	2.0815822	-0.0192723	-0.2509718
4	C	C6	2.4413913	0.8741965	1.2928688
5	H	H13	0.5862006	1.8427590	-0.1938508
6	S	S2	1.0924920	1.7294533	-2.5446275
7	C	C3	-0.5906633	0.2476550	-1.0198776
8	H	H1	-0.4823870	-0.6041174	-1.6975174
9	H	H4	-1.3762737	0.8939814	-1.4201851
10	H	H26	1.5428110	0.9426929	1.9060225
11	H	H27	2.8333997	1.8674831	1.0723040
12	H	H28	3.2078642	0.3006157	1.8132878
13	H	H29	3.3179065	2.1036113	-1.7018721
14	H	H30	2.2441306	3.5018449	-1.3689180
15	H	H31	2.8708098	3.2256421	-3.0091277
16	P	P1	-1.1572646	-0.3208257	0.6126932
17	O	O1	-0.8312264	0.6167188	1.7140108
18	O	O2	-2.7169599	-0.5356794	0.2715331
19	C	C1	-3.5869390	-0.9677243	1.3242083
20	H	H2	-3.3339577	-1.9851230	1.6289888
21	H	H5	-4.5970710	-0.9375793	0.9230193
22	H	H6	-3.5043567	-0.2957566	2.1801133
23	N	N1	-0.6430784	-1.8692459	0.9608546
24	H	H3	0.2102646	-1.8648741	1.5040802
25	C	C2	-0.6916094	-2.9188769	-0.0547641
26	H	H7	-0.6357333	-3.8960124	0.4281068
27	H	H8	0.1222166	-2.8374846	-0.7846116
28	H	H9	-1.6458602	-2.8586407	-0.5816148

AcCysOMe + *N*-methylmaleimide, *RR*

1	C	C	-3.0561790	1.0657938	0.7043866
2	C	C	-3.2952437	-0.7148546	-0.7697553
3	O	O	-3.3129336	1.7683267	1.6426753
4	N	N	-3.8170044	-0.0345435	0.3139800
5	O	O	-3.8166709	-1.6581996	-1.3012279
6	C	C	-5.0435435	-0.4459774	0.9694312
7	H	H	-4.9357664	-1.4603203	1.3542828
8	H	H	-5.8688600	-0.4249058	0.2573161
9	H	H	-5.2293121	0.2498151	1.7852653
10	C	C	-1.9533203	-0.0750392	-1.1198037
11	H	H	-1.9100823	0.0901643	-2.1961482
12	C	C	-1.9160055	1.2107771	-0.2869546
13	H	H	-2.1291205	2.0917491	-0.8988698
14	H	H	-0.9752278	1.4044757	0.2290596
15	S	S	-0.6886206	-1.3174584	-0.6610616
16	C	C	0.8104417	-0.3635912	-1.0869317
17	H	H	0.4948208	0.6240700	-1.4204928
18	H	H	1.3314117	-0.8588777	-1.9082910
19	C	C	1.7646229	-0.1616566	0.1049566
20	H	H	1.1612316	0.0742463	0.9888320
21	N	N	2.7067393	0.9057263	-0.1430051
22	H	H	3.6875941	0.6626283	-0.1445460
23	C	C	2.3009782	2.2039741	-0.0958428
24	O	O	1.1291567	2.5014637	0.0570664
25	C	C	3.3815782	3.2472603	-0.2544977
26	H	H	3.3850831	3.8734423	0.6381861
27	H	H	3.1256980	3.8812322	-1.1036482
28	H	H	4.3725502	2.8197461	-0.4041824
29	C	C	2.5682638	-1.4189410	0.3931285
30	O	O	3.7686354	-1.4565263	0.4633345
31	O	O	1.7771522	-2.4759464	0.5337429
32	C	C	2.4339586	-3.7227293	0.7928620
33	H	H	3.0064651	-3.6579990	1.7176905
34	H	H	3.1029426	-3.9705107	-0.0308829
35	H	H	1.6385663	-4.4568143	0.8799452

AcCysOMe + *N*-methylpropynamide

1	C	C1	0.1524419	-0.8412298	-1.9748661
2	H	H3	-0.6927314	-1.5189001	-1.9043055
3	S	S1	0.4506766	0.1015940	-0.5367788
4	C	C6	-0.9798089	-0.5078314	0.4305990
5	H	H2	-1.0300278	-1.5937013	0.3502753
6	H	H4	-1.9036560	-0.0620964	0.0555833
7	C	C7	-0.8419840	-0.1877182	1.9297044
8	H	H6	0.0813412	-0.6350698	2.3045854
9	N	N2	-1.9722166	-0.7168303	2.6605288
10	H	H9	-2.6831421	-0.0583886	2.9467363
11	C	C8	-2.0697141	-2.0452118	2.9430781
12	O	O3	-1.2275943	-2.8419664	2.5754680
13	C	C9	-3.2789358	-2.4608866	3.7493079
14	H	H12	-2.9322480	-2.8484548	4.7081207
15	H	H13	-3.7816178	-3.2731573	3.2246255
16	H	H14	-3.9818291	-1.6463013	3.9230036
17	C	C10	-0.8140950	1.3130628	2.1598582
18	O	O4	-1.7811046	1.9627148	2.4631237
19	O	O5	0.3947906	1.8144617	1.9486293
20	C	C11	0.4998794	3.2426895	1.9881769
21	H	H10	0.2345532	3.6090717	2.9795201
22	H	H17	-0.1655299	3.6828521	1.2459160
23	H	H18	1.5366722	3.4643079	1.7544207
24	C	C2	0.8566829	-0.8083322	-3.1139777
25	H	H7	0.5595902	-1.4632074	-3.9258761
26	C	C3	2.0235052	0.0785939	-3.2968569
27	O	O1	2.4206074	0.8320146	-2.4219096
28	N	N1	2.6409109	-0.0002165	-4.5083852
29	C	C5	3.7839726	0.8411496	-4.8036130
30	H	H1	3.4988834	1.8956984	-4.8302044
31	H	H5	4.1958010	0.5528035	-5.7698085
32	H	H11	4.5462836	0.7162972	-4.0329680
33	H	H19	2.2596435	-0.5978120	-5.2217119

AcCysOMe + (*N*-methyl)ethynesulfonamide

1	C	C1	3.0477968	-1.1974218	-0.3820934
2	H	H3	4.0710319	-0.8406713	-0.4593704
3	S	S1	2.0367610	-0.4702391	0.8883640
4	C	C6	1.3951501	0.9629602	-0.0662739
5	H	H2	1.2407044	0.6378374	-1.0969137
6	H	H4	2.1177417	1.7774129	-0.0315173
7	C	C7	0.0636918	1.4185245	0.5299963
8	H	H6	0.1740384	1.6591611	1.5908325
9	N	N2	-0.9314387	0.3961213	0.3599475
10	H	H9	-0.7755011	-0.3114269	-0.3486830
11	C	C8	-2.1757301	0.5844387	0.8830649
12	O	O3	-2.4302561	1.5361933	1.5960142
13	C	C9	-3.1917709	-0.4803456	0.5412465
14	H	H12	-4.0680148	0.0065989	0.1127241
15	H	H13	-3.5014523	-0.9747832	1.4639095
16	H	H14	-2.8046174	-1.2201778	-0.1604638
17	C	C10	-0.3515223	2.7099970	-0.1818111
18	O	O4	-1.0910960	2.7677753	-1.1208109
19	O	O5	0.2693741	3.7632741	0.3594989
20	C	C11	-0.0444595	5.0273221	-0.2326674
21	H	H10	0.5172237	5.7662844	0.3321741
22	H	H17	-1.1151906	5.2164715	-0.1585252
23	H	H18	0.2502594	5.0360407	-1.2822429
24	C	C2	2.6454639	-2.1547424	-1.2067904
25	H	H7	3.2882585	-2.5981752	-1.9598103
26	S	S2	0.9963850	-2.8435192	-1.2765631
27	O	O1	0.0354611	-1.7565283	-1.3999027
28	O	O2	1.0815384	-3.8728605	-2.2919653
29	N	N1	0.7534317	-3.5780004	0.1872238
30	H	H5	0.8653830	-4.5797243	0.0733811
31	C	C3	-0.3896558	-3.1738163	1.0150106
32	H	H1	-0.2864332	-3.6792285	1.9747304
33	H	H8	-1.3493748	-3.4347556	0.5628400
34	H	H11	-0.3431812	-2.0999972	1.1854464

AcCysOMe + methyl (*N*-methyl)ethynephosphonamidate, *RS*

1	C	C1	2.0208634	-0.1252035	-0.5895438
2	H	H3	2.9669577	-0.5750923	-0.2974426
3	S	S1	1.4912078	1.2049537	0.4474435
4	C	C6	1.6412350	0.3800200	2.0647623
5	H	H2	1.8659185	1.1498027	2.8017400
6	H	H4	2.4578579	-0.3406609	2.0333618
7	C	C7	0.3374901	-0.3515643	2.4428194
8	H	H6	0.5154007	-0.8470596	3.4035896
9	N	N2	-0.0724674	-1.3280909	1.4672851
10	H	H9	-0.7861361	-1.0686760	0.7852282
11	C	C8	0.6902636	-2.4339616	1.2614882
12	O	O3	1.6841835	-2.6680634	1.9328728
13	C	C9	0.2143891	-3.3612582	0.1694090
14	H	H12	-0.5426136	-2.9002882	-0.4643393
15	H	H13	-0.1970332	-4.2571007	0.6394274
16	H	H14	1.0774695	-3.6642183	-0.4238821
17	C	C10	-0.7877265	0.6506469	2.6268833
18	O	O4	-1.8088547	0.6702266	1.9985959
19	O	O5	-0.4804312	1.5422040	3.5780816
20	C	C11	-1.4670861	2.5494382	3.8168140
21	H	H10	-2.4008889	2.0917831	4.1431355
22	H	H17	-1.6474368	3.1220372	2.9068444
23	H	H18	-1.0567781	3.1849329	4.5966803
24	C	C2	1.3789336	-0.5509709	-1.6783179
25	H	H7	1.8189274	-1.3404680	-2.2803430
26	P	P1	-0.3230790	-0.1177664	-2.0596481
27	O	O1	-1.3380888	-0.7223226	-1.1562729
28	N	N3	-0.5223269	-0.6144351	-3.6321984
29	H	H1	-1.4668632	-0.9167253	-3.8339087
30	C	C3	0.1680089	0.0681488	-4.7252424
31	H	H5	1.2141670	0.2173501	-4.4486053
32	H	H8	-0.2638139	1.0463851	-4.9535043
33	H	H11	0.1440078	-0.5583267	-5.6166196
34	O	O2	-0.3286197	1.4924619	-2.0501762
35	C	C4	-1.3632270	2.2031508	-1.3521999
36	H	H15	-2.2852307	2.1892090	-1.9360405
37	H	H16	-1.0075604	3.2254779	-1.2436636
38	H	H19	-1.5410192	1.7540239	-0.3745137

AcCysOMe + methyl (*N*-methyl)ethynephosphonamidate, *RR*

1	C	C1	1.8875557	-0.1006031	-0.5112607
2	H	H3	2.7600198	-0.7169873	-0.3023239
3	S	S1	1.5126459	1.0903661	0.7381695
4	C	C6	1.5632292	-0.0142350	2.1873886
5	H	H2	1.8955981	0.5757543	3.0400416
6	H	H4	2.2748383	-0.8191139	2.0019209
7	C	C7	0.1774950	-0.6259005	2.4672374
8	H	H6	0.3054746	-1.3453438	3.2844039
9	N	N2	-0.3869356	-1.3029781	1.3279679
10	H	H9	-1.0470947	-0.7899827	0.7422752
11	C	C8	0.1888796	-2.4399156	0.8640063
12	O	O3	1.1409381	-2.9627472	1.4239957
13	C	C9	-0.4388630	-3.0325429	-0.3757737
14	H	H12	-1.1923209	-2.3772287	-0.8130573
15	H	H13	-0.8904070	-3.9884814	-0.1040164
16	H	H14	0.3547400	-3.2337146	-1.0969763
17	C	C10	-0.8130757	0.4281441	2.9352864
18	O	O4	-1.9057543	0.6009976	2.4778242
19	O	O5	-0.3047633	1.1476527	3.9464524
20	C	C11	-1.1669725	2.1642783	4.4643885
21	H	H10	-2.0871510	1.7205231	4.8449367
22	H	H17	-1.4128007	2.8820600	3.6814672
23	H	H18	-0.6106437	2.6429025	5.2657413
24	C	C2	1.2222150	-0.2387587	-1.6603904
25	H	H7	1.5940207	-0.9441555	-2.3987033
26	N	N1	-0.1493237	2.1637305	-2.0070683
27	C	C5	0.7486816	2.8154552	-2.9552588
28	H	H1	0.9810354	3.8172890	-2.5967913
29	H	H5	0.3260341	2.8744390	-3.9619822
30	H	H11	1.6840480	2.2527380	-3.0039686
31	H	H19	-0.9631203	2.6885014	-1.7151451
32	P	P1	-0.3729454	0.5323982	-1.9848285
33	O	O1	-1.4606469	0.0837955	-1.0784008
34	O	O2	-0.6276502	0.1373051	-3.5340443
35	C	C3	-1.1067248	-1.1747687	-3.8326471
36	H	H15	-0.3792800	-1.9346446	-3.5302105
37	H	H20	-1.2481237	-1.2201347	-4.9100608
38	H	H21	-2.0528518	-1.3560936	-3.3205951

Experimental (for general methods, see Ref S6)

NMR spectra of 1·D

Methyl *N*-acetyl-*S*-[(3*RS*)-1-phenylmethyl-2,5-dioxopyrrolidin-3-yl]-L-cysteinate, or methyl *N*-acetyl-*S*-[(3*RS*)-1-benzylmale-imid-4-yl]-L-cysteinate, an almost 1:1 mixture of two diastereomers (**1**), was prepared from commercially available *N*-benzylmaleimide, as reported.[S5] The addition of a solution of Na_2HPO_4 in D_2O to **1** dissolved in DMSO, quenching with Me_3SiCl/CD_3OD, and partition between water and dichloromethane, gave rise to **1**·D, where the signals of H3', at 3.93 ppm, disappeared and the $^{13}C\{^1H\}$ signals of C3' at 40.1 and 38.6 ppm changed as expected.

The reaction was also followed by NMR, in DMSO-d_6, by addition of a drop of a solution of K_3PO_4 in D_2O (Scheme 5 of the main text); the D/H exchange was quick.

^{1}H NMR spectrum of **1** ($CDCl_3$, 400 MHz)

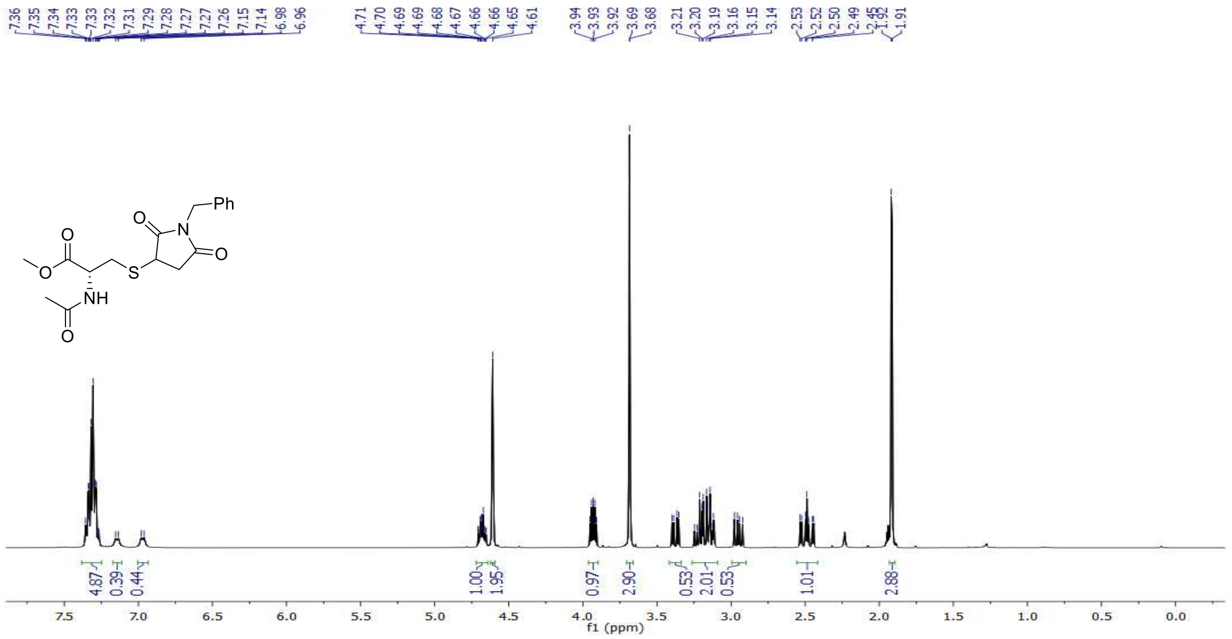

^{1}H NMR spectrum of **1**·D ($CDCl_3$, 400 MHz)

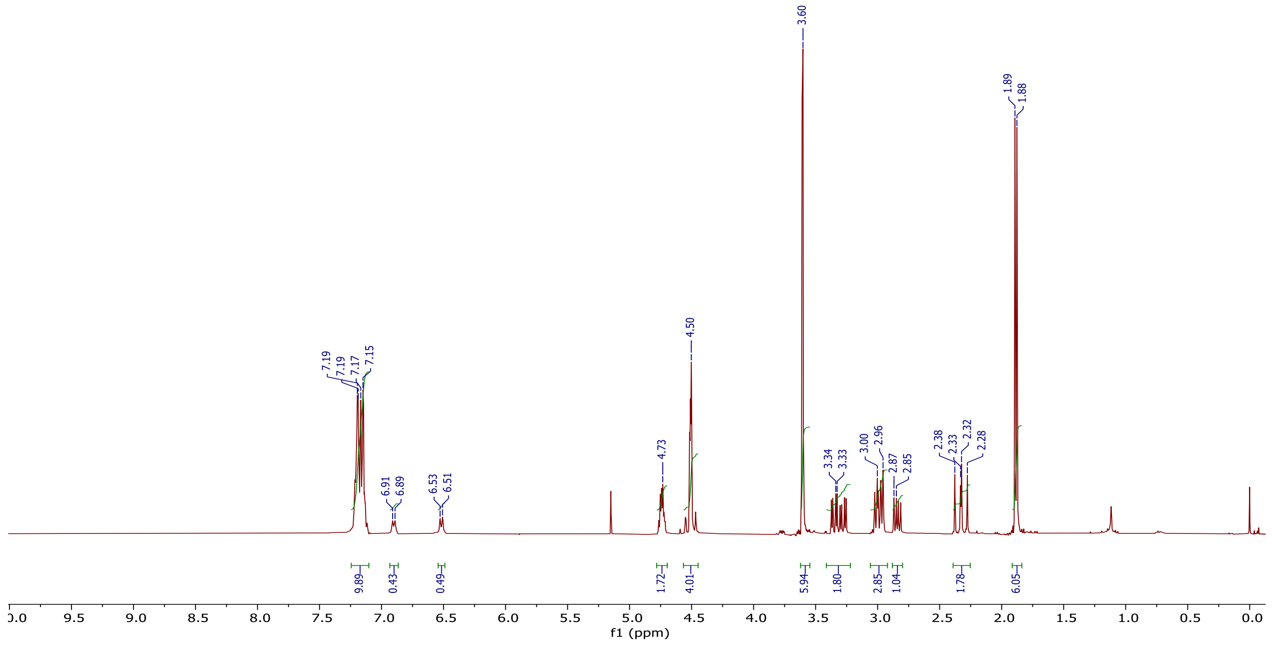

^{13}C NMR spectrum of **1·**D ($CDCl_3$, 100.6 MHz)

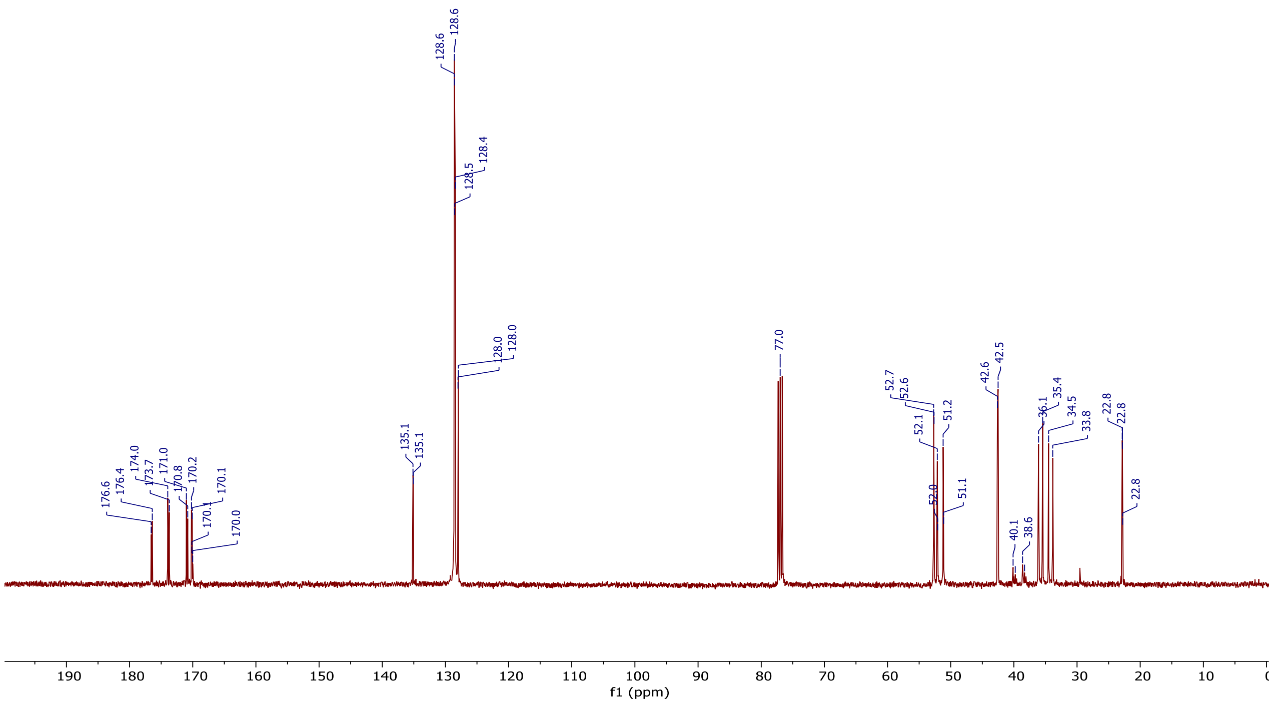

Treatment of 1 with NaH

Sodium hydride (60% dispersion in mineral oil, ca. 4.00 mg, 0.10 mmol, 0.8 equiv) was added to a solution of **1** (47 mg, 0.13 mmol) in CD_3CN (1.0 mL) (Scheme 5 of the main text). The solution turned dark red. Within 10 min its ^1H NMR spectrum was registered, which was compared with that of the starting material (**1**). The signal at 3.95 ppm (two overlapped dd corresponding to the two diastereomers of **1**) quickly and significantly diminished, but also some percentage of deprotonation of the signals at 2.50 ppm (succinimide methylene) was noted. Although not indicated in the drawings, the NH protons of the acetamido groups of the two diastereomers also disappeared partially.

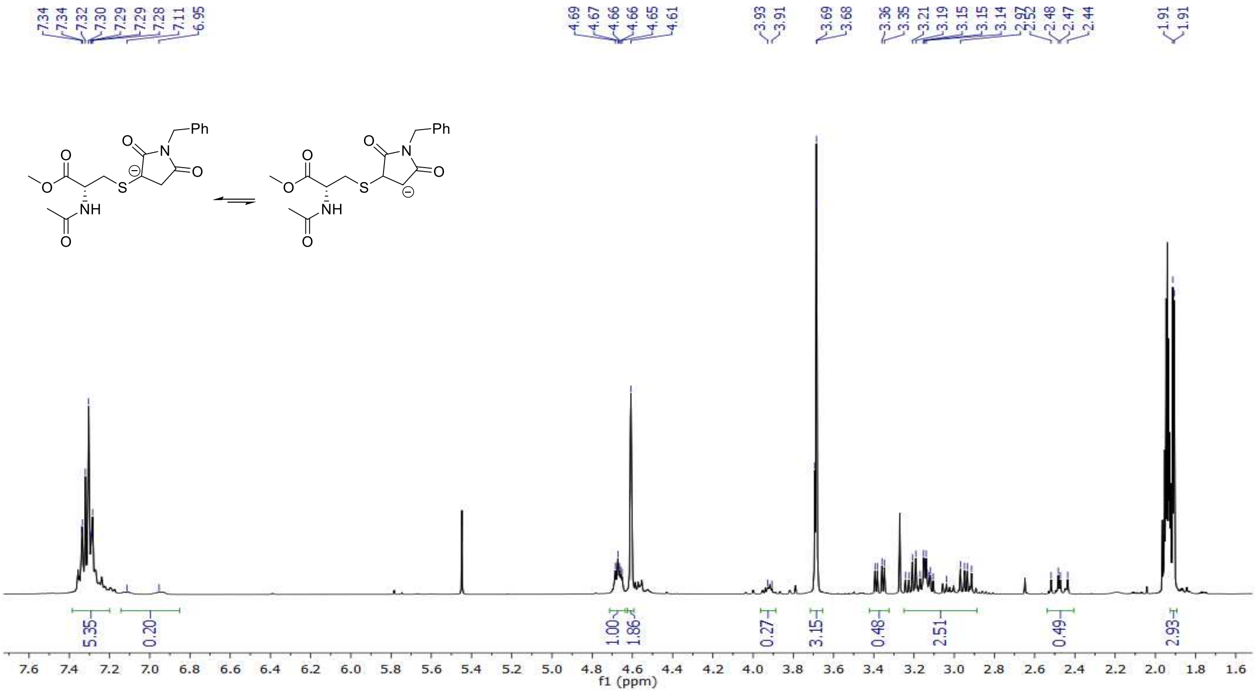

Comparison of the ^{1}H NMR spectrum of **1** in CD_3CN before and after addition of NaH:

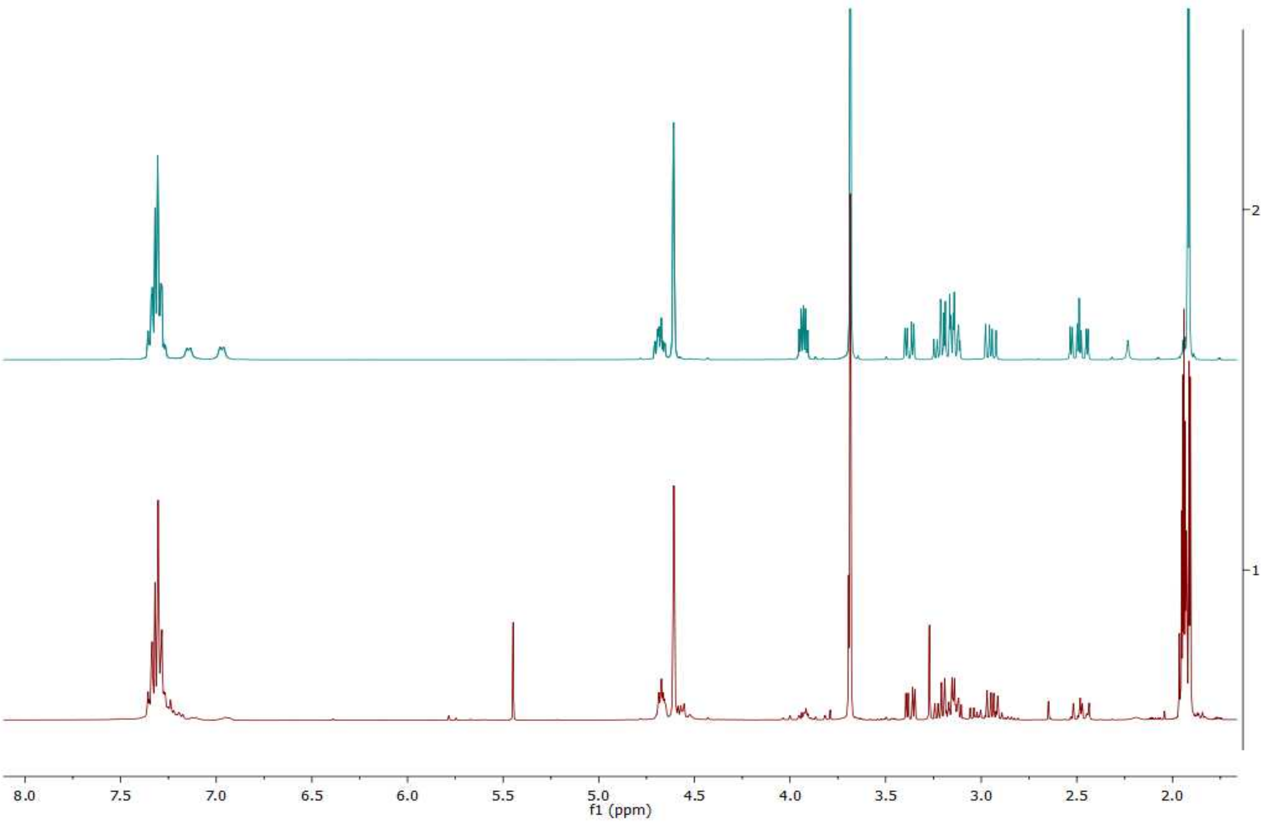

Migration of AcCysOMe from 1 to 2

tert-Butyllithium (1.7 M in pentane, 16 µL, 27 µmol, 0.1 equiv) was added to a solution of **1** (98 mg, 0.27 mmol) and *N*-benzylpropynamide (**2**, 43 mg, 0.27 mmol) in anhydrous THF (3.0 mL) under N_2 atmosphere, at –78 °C. The reaction was then stirred at room temperature for 2 h. After neutralization with aqueous phosphate buffer, extraction with dichloromethane (x 3), and drying of the combined organic layers, the organic solvents were removed under reduced pressure (rotary evaporator). The ^{1}H NMR spectrum of the crude mixture was registered in $CDCl_3$.

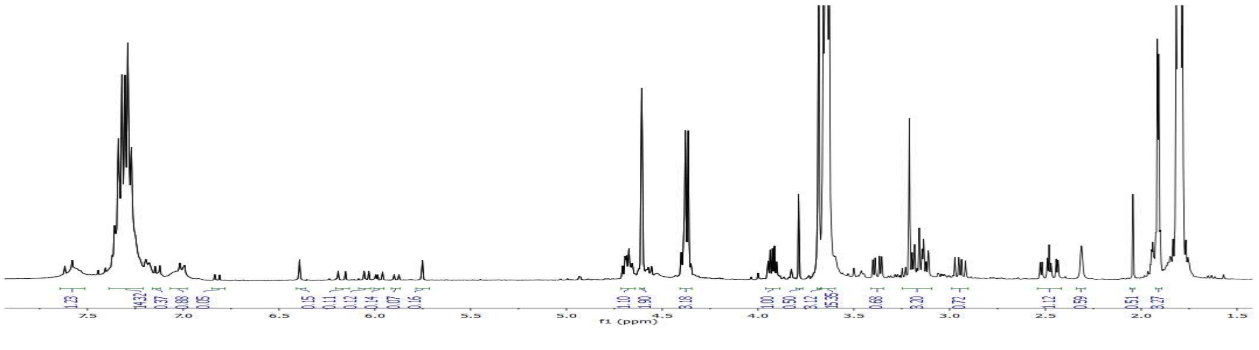

Double bonds (of known[S5] AcCysOMe(S)–CH=CH–CONHBn, *Z*-**3** + *E*-**3**, around 10%) are observed. Also the signal of the double bond of *N*-benzylmaleimide (at 6.45 ppm) appeared, in 5–10%. The formation of these compounds was confirmed by TLC and MS.

tert-Butyllithium (1.7 M, 165 µL, 280 µmol, 0.85 equiv) was added to a solution of **1** (120 mg, ca. 0.33 mmol) and **2** (52 mg, 0.33 mmol) in anhydrous THF (3.5 mL) under N_2 atmosphere, at –78 °C. The solution, which immediately turned dark red, was stirred for 2 h at room temperature. After neutralization, extraction with CH_2Cl_2, and drying of the combined organic solutions, the solvent was removed under reduced pressure. The residue was chromatographically compared with pure samples of the expected products and was analyzed by ^{1}H NMR spectroscopy to contain *Z*-**3** + *E*-**3** (ca. 70%) and polymeric material rather than *N*-benzylmaleimide.

Exchange of thiolates in the maleimide adducts (mechanisms)

Formally, an exchange of MeSH between the methylsulfanylsuccinimide shown in Scheme S1 and any propynamide is feasible. The hypothetical equilibrium must be shifted very far to the right; it will suffice to find a suitable catalyst and/or to reach the appropriate temperature.

In vitro, when succinimide **1** and *N*-benzylpropynamide (**2**) were mixed in CH_3CN at rt plus a catalytic amount of Na^+DMSO^- or in THF at –78 ºC plus ^{t}BuLi (either in catalytic or substoichiometric amounts), the formation of **3** (*Z/E* mixture) was rapidly and clearly observed by TLC and NMR. The exchange takes place, in agreement with the predictions, but in the absence of a source of protons it cannot be avoided that the generated allenolate-type ion, during the workup, gives rise to the *Z/E* mixture instead of the *Z* isomer. The coproduct from the exchange, *N*-benzylmaleimide, could not be isolated as it undergoes a rapid polymerization (formation of a dark red polymer) in non-aqueous basic media.[S7]

Scheme S1. Plausible exchange reaction on the basis of M06-2X calculations. Δ*E* and Δ*G*˚ values in kcal/mol, in bold. Reaction of **1** and **2** in the presence of strong bases

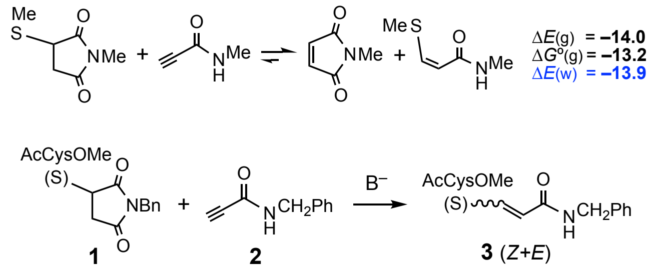

If the elimination of RS^- (E1cB, from the minor succinimide anion with the negative charge at C4, see Scheme 4) or of RSH (E2, from the neutral adduct, under basic catalysis) occurs, the generated species, RS^-, will immediately react with the ynamide. In aqueous solutions, this reversal or retro-Michael reactions is assumed for the exchange of thiols in Cys–maleimide-containing ADCs. As shown in Scheme S2, there is a series of equilibrium reactions with very small barriers (as predicted in Scheme 4).

Scheme S2. Exchange reactions via elimination–addition steps

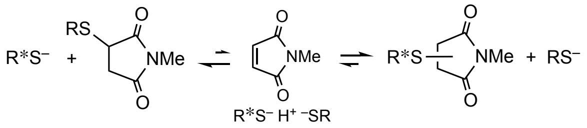

Other reactions of alkylsulfanyl derivatives are possible. When the acceptors are strong, as in the case of 4-phenyl-1,2,4-triazoline-3,5-dione (PTAD), and the new bond (S–N) is weaker than a standard S–C bond, it is known that a second unity of thiol or thiolate causes the formation of dialkyl disulfide,[S8] as shown in Scheme 12.

We have calculated the energies of these species for the case of the methanethiolate ion (MeS^-). The overall reaction is highly exothermic (Scheme S3), as are all the individual steps. In contrast, for the parallel reaction of MeS^- with the adduct from *N*-methylmaleimide, the reaction cannot work: the value of Δ$E_{(g)}$ was 3.7 kcal/mol and that of Δ$E_{(w)}$ 18.7 kcal/mol (both positive).

Scheme S3. Known reaction of dimerization of thiols, mediated by very strong acceptors, which probably pass through the Michael adduct, followed by the formation of the S–S bond. Values of Δ*E* in kcal/mol

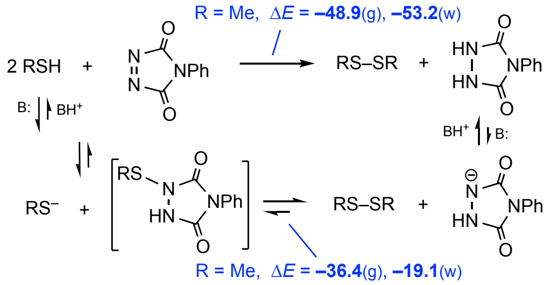

Another possibility is that a S_N2 mechanism is involved, such as the identity reaction[S9] depicted in Scheme S4, where the substitution would be relatively favored as it occurs at position a to a carbonyl group. We investigated the TS for such a simple S_N2 reaction (Scheme S4, with R = R* = Me). With M06-2X, in the gas phase, the energy of the located TS was around 3 kcal/mol above the sum of the energies of the individual components (quite a low barrier), whereas in water it was 26 kcal/mol above them (quite a high barrier). The nature of the TS, which is much less polar than MeS^-, explains this large effect of the solvent polarity. With larger thiolate ions the effect of water is expected to be less significant.

Scheme S4. Alternative mechanism (a standard S_N2) for the exchange of thiols

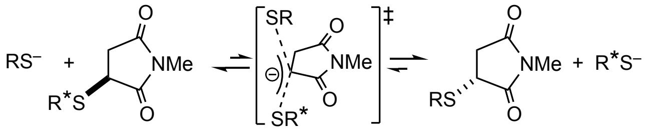

Attempted experiments to gain more insight into the exchange mechanisms in vitro, with partially deuterated adducts and lithium 1-dodecanethiolate, were not conclusive. The known tendency of succinimide intermediates to undergo ring opening in basic aqueous media and of maleimides to undergo anionic polymerization in non-protic media were prevalent.

References

(S1) Gaussian 16, Revision C.01, https://gaussian.com. Frisch, M. J.; Trucks, G. W.; Schlegel, H. B.; Scuseria, G. E.; Robb, M. A.; Cheeseman, J. R.; Scalmani, G.; Barone, V.; Petersson, G. A.; Nakatsuji, H.; Li, X.; Caricato, M.; Marenich, A. V.; Bloino, J.; Janesko, B. G.; Gomperts, R.; Mennucci, B.; Hratchian, H. P.; Ortiz, J. V.; Izmaylov, A. F.; Sonnenberg, J. L.; Williams-Young, D.; Ding, F.; Lipparini, F.; Egidi, F.; Goings, J.; Peng, B.; Petrone, A.; Henderson, T.; Ranasinghe, D.; Zakrzewski, V. G.; Gao, J.; Rega, N.; Zheng, G.; Liang, W.; Hada, M.; Ehara, M.; Toyota, K.; Fukuda, R.; Hasegawa, J.; Ishida, M.; Nakajima, T.; Honda, Y.; Kitao, O.; Nakai, H.; Vreven, T.; Throssell, K.; Montgomery, J. A., Jr.; Peralta, J. E.; Ogliaro, F.; Bearpark, M. J.; Heyd, J. J.; Brothers, E. N.; Kudin, K. N.; Staroverov, V. N.; Keith, T. A.; Kobayashi, R.; Normand, J.; Raghavachari, K.; Rendell, A. P.; Burant, J. C.; Iyengar, S. S.; Tomasi, J.; Cossi, M.; Millam, J. M.; Klene, M.; Adamo, C.; Cammi, R.; Ochterski, J. W.; Martin, R. L.; Morokuma, K.; Farkas, O.; Foresman, J. B.; Fox, D. J. Gaussian, Inc., Wallingford CT, 2016.

(S2) (a) Spartan'18.2, https://www.wavefun.com. Deppmeier, B.; Driessen, A.; Hehre, T.; Hehre, W.; Klunzinger, P.; Ohlinger, S.; Schnitker, J. Wavefunction, Inc., 18401 Von Karman Avenue, Irvine, CA 92612. (b) ORCA 4.2.1, https://orcaforum.kofo.mpg.de/-app.php/portal. Neese, F.; Wennmohs, F.; Becker, U.; Riplinger. C. The ORCA Quantum Chemistry Program Package. *J. Chem. Phys.* **2020**, *152*, 224108-1–224108-18.

(S3) (a) Zhao, Y.; Truhlar, D. G. *Theor. Chem. Acc.* **2008**, *120*, 215−241. (b) Zhao, Y.; Truhlar, D. G. *Acc. Chem. Res.* **2008**, *41*, 157−167.

(S4) (a) Castro-Alvarez, A.; Carneros, H.; Sánchez, D.; Vilarrasa, J. *J. Org. Chem.* **2015**, *80*, 11977–11985. (b) Castro-Alvarez, A.; Carneros, H.; Calafat, J.; Costa, A. M.; Marco, C.; Vilarrasa, J. *ACS Omega* **2019**, *4*, 18167−18194.

(S5) https://cccbdb.nist.gov/vibscalejust.asp

(S6) Petit, E.; Bosch, L.; Costa, A. M.; Vilarrasa, J. *J. Org. Chem*. **2019**, *84*, 11170−11176.

(S7) (a) Oishi, T.; Onimura, K.; Isobe, Y.; Yanagihara, H.; Tsutsumi, H. *J. Polym. Sci. Part A: Polym. C* **2000**, *38*, 310–320 (and references therein). For very recent reviews, see: (b) Oz, Y.; Sanyal, A. *Chem. Rec.* **2018**, *18*, 570–586. (c) Dolci, E.; Froidevaux, V.; Joly-Duhamel, C.; Auvergne, R.; Boutevin, B.; Caillol, S. *Polym. Rev.* **2016**, *56*, 512–556.

(S8) The reaction works with DEAD and related azo derivatives conjugated with EWGs, including PTAD: (a) Harasawa, S.; Yoshida, K.; Kojima, C.; Araki, L.; Kurihara, T. *Tetrahedron* **2004**, *60*, 11911–11922. (b) Christoforou, A.; Nicolaou, G.; Elemes, Y. *Tetrahedron Lett.* **2006**, *47*, 9211–9213. (c) Morais, G. B.; Falconer, R. A. *Tetrahedron Lett.* **2007**, *48*, 7637–7641. For reviews of the formation of RSSR', see: (d) Mandal, B.; Basu, B. *RSC Adv*. **2014**, *4*, 13854–13881. (e) Wang, M.; Jiang, X. *Top. Curr. Chem*. **2018**, *376*, 1–40. For the addition of MeSH to dimethyl azodicarboxylate, our M06-2X calculations predict $\Delta E_{(g)}$ = –34.5 kcal/mol and $\Delta E_{(w)}$ = –38 kcal/mol; for that of MeS^-, $\Delta E_{(g)}$ = –41 kcal/mol and $\Delta E_{(w)}$ = –30 kcal/mol; these last values are lower than those of PTAD (Scheme S3).

(S9) (a) Gonzales, J. M.; Allen, W. D.; Schaefer III, H. F. *J. Phys. Chem. A* **2005**, *109*, 10613–10628. (b) Hoz, S.; Basch, H.; Wolk, J. L.; Hoz, T.; Rozental, E. *J. Am. Chem. Soc.* **1999**, *121*, 7724–7725. (c) Shaik, S. S. *J. Am. Chem. Soc.* **1984**, *106*, 1227–1232.
